# Snf1-related kinase improves cardiac mitochondrial efficiency and decreases mitochondrial uncoupling

**DOI:** 10.1038/ncomms14095

**Published:** 2017-01-24

**Authors:** Amy K. Rines, Hsiang-Chun Chang, Rongxue Wu, Tatsuya Sato, Arineh Khechaduri, Hidemichi Kouzu, Jason Shapiro, Meng Shang, Michael A. Burke, Eltyeb Abdelwahid, Xinghang Jiang, Chunlei Chen, Tenley A. Rawlings, Gary D. Lopaschuk, Paul T. Schumacker, E. Dale Abel, Hossein Ardehali

**Affiliations:** 1 Feinberg Cardiovascular Research Institute, Feinberg School of Medicine, Northwestern University, Chicago, Illinois 60611, USA; 2 Division of Endocrinology, Metabolism, and Diabetes and Program in Molecular Medicine, University of Utah, School of Medicine, Salt Lake City, Utah 84132, USA; 3 Cardiovascular Research Centre, Mazankowski Alberta Heart Institute, University of Alberta, Edmonton, Alberta, Canada T6G 2B7

## Abstract

Ischaemic heart disease limits oxygen and metabolic substrate availability to the heart, resulting in tissue death. Here, we demonstrate that the AMP-activated protein kinase (AMPK)-related protein Snf1-related kinase (SNRK) decreases cardiac metabolic substrate usage and mitochondrial uncoupling, and protects against ischaemia/reperfusion. Hearts from transgenic mice overexpressing SNRK have decreased glucose and palmitate metabolism and oxygen consumption, but maintained power and function. They also exhibit decreased uncoupling protein 3 (UCP3) and mitochondrial uncoupling. Conversely, *Snrk* knockout mouse hearts have increased glucose and palmitate oxidation and UCP3. SNRK knockdown in cardiac cells decreases mitochondrial efficiency, which is abolished with UCP3 knockdown. We show that Tribbles homologue 3 (Trib3) binds to SNRK, and downregulates UCP3 through PPARα. Finally, SNRK is increased in cardiomyopathy patients, and SNRK reduces infarct size after ischaemia/reperfusion. SNRK also decreases cardiac cell death in a UCP3-dependent manner. Our results suggest that SNRK improves cardiac mitochondrial efficiency and ischaemic protection.

## Abstract (web summary)

The Snf1-related kinase (SNRK) is widely expressed and yet its function is poorly understood. Here the authors show that SNRK regulates mitochondrial coupling via the Trib3-PPARα-UCP3 pathway and that cardiac overexpression of SNRK decreases metabolic substrate usage and oxygen consumption but maintains cardiac function and energy in mice.

Ischaemic heart disease is a leading cause of death worldwide. As ischaemia restricts blood flow to the heart, cardiac tissue is damaged due to the reduced availability of oxygen and metabolic substrates. Interventions to increase non-oxidative glycolytic metabolism have exhibited some success as a clinical therapy for ischaemic heart disease^1^,^2^. However, no protein pathways or pharmacological agents are known to lower metabolic substrate usage and oxygen consumption while maintaining normal cardiac work and function. Identification of novel proteins and pathways that modulate substrate metabolism to improve energy production from limited oxygen and substrate availability could potentially lead to new therapies aimed to increase metabolic efficiency and decrease tissue death during heart disease.

Sucrose nonfermenting 1 (Snf1)-related kinase (SNRK) is a serine/threonine kinase and member of the AMP-activated protein kinase (AMPK) family. AMPK has been studied extensively as a master metabolic regulator^3^, but little is known about the cellular role of SNRK. SNRK mRNA is a broadly expressed^4^,^5^,^6^ monomeric protein that is activated by phosphorylation on its conserved T-loop residue by liver kinase B1 (LKB1), an activator of other AMPK family members^7^,^8^. Unlike some other AMPK-related kinases, SNRK does not need additional subunits or activating stimuli, such as an increase in the AMP:ATP ratio, to be activated by LKB1 (ref. 7). SNRK levels are induced in response to apoptosis in rat granular neurons^6^, and SNRK suppresses adipocyte inflammation^9^ and is necessary for proper arterial/vein specification in zebrafish^10^.

Previously, we demonstrated that SNRK reduces the proliferation of colorectal cancer cells, and gene array analysis suggested that SNRK may also regulate genes involved in metabolic processes^11^. Recently, global SNRK homozygous knockout (KO) was found to cause lethality within 24 h of birth, with cardiomyocyte-specific homozygous KO causing death by 8–10 months of age^12^. Studies of the global KO neonates at age of lethality demonstrated that there were broad changes in metabolic gene expression, and cardiomyocyte-specific KO neonatal mice had altered fatty acid staining, further indicating that SNRK may play a role in metabolic processes. However, information about the functional and mechanistic role of SNRK remains limited. In this study, we investigated whether SNRK specifically regulates cardiac metabolism, and the mechanisms for this function. Using SNRK transgenic and KO mouse models, we found that SNRK decreases cardiac metabolic substrate usage and mitochondrial coupling, while protecting against ischaemia/reperfusion injury. Mechanistically, the effects on substrate usage and cell death are dependent on UCP3, which is downregulated through suppression of PPARα by Trib3, a novel binding partner of SNRK.

## Results

### SNRK TG mice have decreased metabolic substrate usage

**Figure 1:**
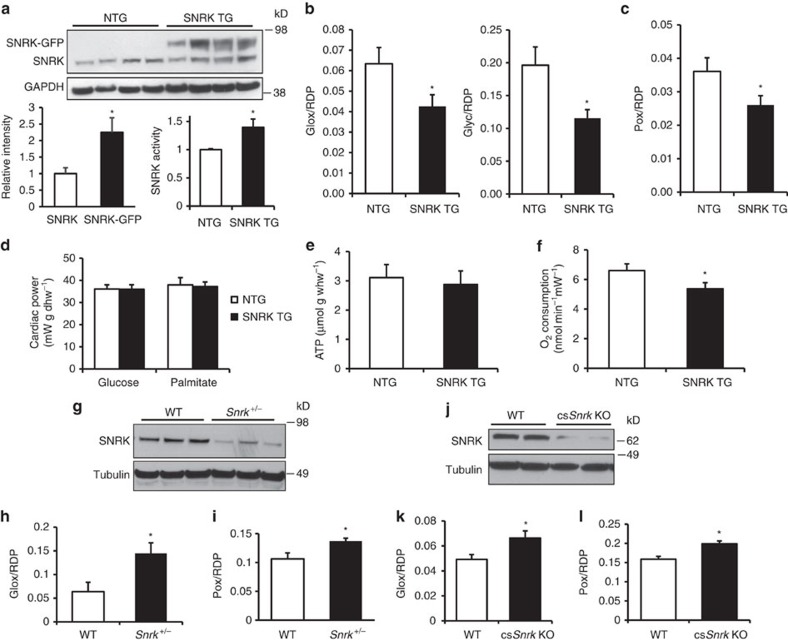
SNRK decreases glucose and palmitate oxidation, but not cardiac function. (**a**, top) Endogenous SNRK and SNRK-GFP transgene protein levels in NTG and SNRK TG mouse hearts using SNRK antibody. (bottom left) Quantification of endogenous SNRK and transgenic SNRK-GFP expression in the transgenic mice. (bottom right) Activity levels with H3.3 substrate peptide from whole-cell lysates of NTG and SNRK TG mouse hearts. Signal was normalized to background activity measured without addition of H3.3; *n*=3. (**b**) Glucose oxidation (Glox) and glycolysis (Glyc) normalized to rate-developed pressure product (RDP) in perfused working hearts from NTG and SNRK TG mice. *n*=6. (**c**) Palmitate oxidation (Pox) normalized to RDP in working hearts from NTG and SNRK TG mice. *n*=6. (**d**) Cardiac power measurements in NTG and SNRK TG hearts perfused with both glucose and palmitate, along with [5-^3^H]glucose and [U-^14^C]glucose (labelled glucose) or [9,10-^3^H]palmitate (labelled palmitate). (**e**) ATP levels in NTG and SNRK TG working hearts. *n*=6, whw=wet heart weight. (**f**) Oxygen consumption in NTG and SNRK TG working hearts normalized to cardiac power. *n*=6. (**g**) SNRK protein levels in WT and *Snrk*^+/−^ hearts, representative of 4–5 independent samples. (**h**) Glox normalized to RDP in perfused working hearts from WT and *Snrk*^+/−^ mice. *n*=5 for WT, *n*=4 for SNRK^+/−^. (**i**) Pox normalized to RDP in working hearts from WT and *Snrk*^+/−^ mice. (**j**) SNRK protein levels in WT and cs*Snrk* KO mice, representative of three independent samples. (**k**) Glox normalized to RDP in perfused working hearts from WT and cs*Snrk* KO mice. *n*=9 for WT, *n*=10 for cs*Snrk* KO. (**l**) Pox normalized to RDP in perfused working hearts from WT and cs*Snrk* KO mice. Data are represented as mean±s.e.m. *P≤0.05 by Student’s *t*-test.

Using gene array analysis, we previously demonstrated that SNRK overexpression alters genes involved in cellular metabolism^11^, and neonatal homozygous *Snrk* KO mice were shown to have altered metabolic gene expression^12^. In addition, AMPK is a major regulator of cardiac energy metabolism^13^. Thus, we hypothesized that SNRK regulates cardiac energy metabolism. To investigate this hypothesis, we generated mice with moderate transgenic (TG) overexpression of SNRK using the alpha-myosin heavy chain (αMHC) promoter, which restricts expression to cardiomyocytes^14^. We demonstrated that TG SNRK protein is expressed twofold relative to endogenous cardiac SNRK using a SNRK antibody (Fig. 1a). The overexpression was confirmed by measuring SNRK mRNA levels and additional Western blotting (Supplementary Fig. 1a). We also verified that the transgene is expressed only in the heart (Supplementary Fig. 1b).

The SNRK TG protein increased the kinase activity of heart lysates in the presence of the known SNRK substrate peptide histone H3.3 (ref. 10) by ∼40% (Fig. 1a). Certain other kinases can phosphorylate H3.3 and contribute to background signal, but the increase in activity relative to the nontransgenic (NTG) hearts is attributable to the expression of the TG SNRK. Immunoprecipitated SNRK-GFP protein is also functional, as it is active in phosphorylating the H3.3 peptide (Supplementary Fig. 1c). Thus, the TG SNRK mouse model has moderate cardiac overexpression of active SNRK kinase.

SNRK TG mice were born at expected Mendelian ratios. Body weight and heart weight/body weight of SNRK TG mice were similar to their NTG littermates (Supplementary Table 1). SNRK TG mice also had normal cardiac function, as measured by ejection fraction (EF) and fractional shortening (FS, Supplementary Fig. 1e). Other echocardiographic parameters, including interventricular septum (IVS) thickness, left ventricular posterior wall thickness, left ventricular internal dimension and left ventricular volume were also unchanged (Supplementary Table 2).

We next measured glucose and fatty acid metabolism in the hearts of SNRK TG mice to determine the impact of SNRK overexpression on cardiac energy metabolism. These studies were performed in isolated, working hearts perfused with radioactively labelled glucose and palmitate. SNRK TG hearts exhibited decreased glycolysis and glucose oxidation relative to cardiac power compared with their NTG littermates (Fig. 1b), indicating reduced utilization of glucose as an energetic substrate. Palmitate oxidation was also decreased in SNRK TG hearts (Fig. 1c), demonstrating reduced utilization of fatty acids. Despite a decrease in energetic substrate usage, cardiac power was unchanged in these hearts (Fig. 1d), and ATP levels were also maintained in the hearts perfused with glucose or palmitate (Fig. 1e). ATP production was also unchanged in HL1 cardiomyocytes with SNRK overexpression (Supplementary Fig. 1d). Functional parameters, such as heart rate, developed pressure, and cardiac output, were unchanged in the working hearts (Supplementary Table 1). Maintenance of cardiac energy was further supported by a lack of activation of phosphorylated AMPK in the SNRK TG hearts relative to NTG hearts (Supplementary Fig. 2a). Cardiac oxygen consumption in the working hearts was also decreased in the SNRK TG mice (Fig. 1f). Endogenous substrates were not depleted, as demonstrated by measurements of triglyceride and glycogen stores in SNRK TG hearts (Supplementary Fig. 2c,d). Thus, SNRK TG hearts exhibited decreased utilization of both glucose and palmitate and decreased oxygen consumption, but maintenance of cardiac performance and energy.

We also generated heterozygous *Snrk* KO mice, since homozygous *Snrk* KO was lethal within 24 h of birth^12^. Heterozygous KO led to an ∼50% decrease in SNRK expression in the heart (Fig. 1g) without a change in heart or body size (Supplementary Table 3). Cardiac performance was also unchanged in these mice (Supplementary Fig. 1f and Supplementary Table 4). Opposite to SNRK TG mice, working hearts from the *Snrk*^+/−^ mice had increased glucose oxidation (Fig. 1h) and palmitate oxidation (Fig. 1i) without a change in heart rate or other functional parameters (Supplementary Table 3). Phosphorylated AMPK was also not altered in these hearts (Supplementary Fig. 2b), or in HL1 cardiomyocytes with SNRK knockdown (Supplementary Fig. 2e). To further demonstrate that the function of SNRK in cardiomyocytes is responsible for its effect on heart metabolism, we generated cardiac-specific *Snrk* KO (cs*Snrk* KO) mice by crossing *Snrk* floxed mice with αMHC-Cre mice. These mice had loss of SNRK protein in cardiomyocytes (Fig. 1j) without altered cardiac performance (Supplementary Fig. 1g and Supplementary Table 5). Similar to the *Snrk*^+/−^ mice, working hearts from the cs*Snrk* KO mice exhibited increased glucose and palmitate oxidation (Fig. 1k,l), and also had a small decrease in aortic systolic and developed pressure (Supplementary Table 6). These results demonstrate that a reduction in SNRK causes an increase in substrate flux in the heart.

### SNRK TG mice have increased mitochondrial coupling

**Figure 2:**
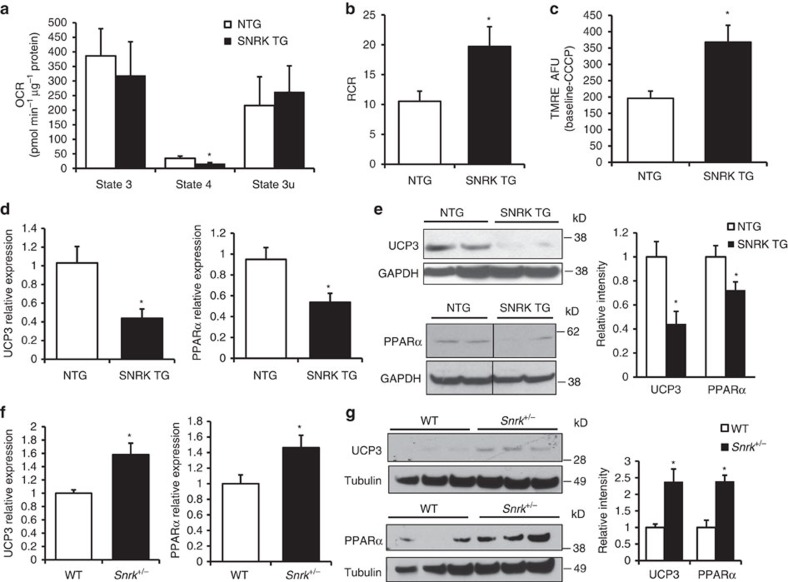
SNRK increases mitochondrial coupling and decreases UCP3 and PPARα. (**a**) Oxygen consumption rate (OCR) with pyruvate and malate in mitochondria from NTG and SNRK TG mouse hearts. State 3 respiration indicates oxygen consumption with ADP, state 4 indicates respiration with oligomycin, and state 3u indicates uncoupled respiration with CCCP. *n*=3. (**b**) Respiratory control ratio (RCR, state 3/state 4 respiration) in mitochondria from NTG and SNRK TG mouse hearts. (**c**) Mitochondrial membrane potential in cardiomyocytes from NTG and SNRK TG mice. AFU=arbitrary fluorescence units. *n*=6. (**d**) UCP3 (*n*=6) and PPARα (*n*=9) mRNA levels in NTG and SNRK TG mouse hearts. (**e**) UCP3 and PPARα protein levels in NTG and SNRK TG mouse hearts. Graphs show summary of *n*=7–8 for UCP3 relative to GAPDH, and *n*=7–8 for PPARα relative to tubulin. (**f**) UCP3 and PPARα mRNA levels in WT and *Snrk*^+/−^ mouse hearts. *n*=3. (**g**) UCP3 and PPARα protein levels in WT and *Snrk*^+/−^ mouse hearts, representative of 7–8 independent samples summarized in densitometry graph. Data are represented as mean±s.e.m. *P≤0.05 by Student’s *t*-test.

To investigate the mechanism for decreased metabolic substrate usage, we measured the function of cardiac mitochondria isolated from SNRK TG mice. State 4 oxygen consumption in the presence of oligomycin with pyruvate and malate substrates was lower in mitochondria isolated from SNRK TG hearts than from NTG hearts (Fig. 2a), demonstrating that SNRK TG mitochondria have decreased levels of uncoupled respiration. Moreover, SNRK TG mitochondria had an increased respiratory control ratio (RCR, state 3 respiration/state 4 respiration), consistent with increased mitochondrial coupling (Fig. 2b). In cardiomyocytes isolated from SNRK TG hearts, the mitochondrial membrane potential was increased compared with NTG controls (Fig. 2c). In addition, SNRK TG hearts had decreased mRNA levels (Fig. 2d) and protein levels of uncoupling protein 3 (UCP3, Fig. 2e). This decrease in UCP3 is predicted to increase mitochondrial coupling^15^,^16^. UCP2 protein levels were unchanged in the TG hearts (Supplementary Fig. 2f). We also observed that the expression of PPARα, a transcriptional regulator of UCP3 (ref. 17), was decreased in the SNRK TG hearts both at the mRNA (Fig. 2d) and protein levels (Fig. 2e). Conversely, expression of UCP3 and PPARα was increased in *Snrk*^+/−^ hearts on the mRNA (Fig. 2f) and protein levels (Fig. 2g). PPARα target genes were also decreased in SNRK TG hearts (Supplementary Fig. 3g) and increased in *Snrk* KO hearts (Supplementary Fig. 3h).

SNRK TG hearts had no change in mitochondrial complex activity (Supplementary Fig. 4a), and the TG and KO hearts displayed no change in mitochondrial content (Supplementary Fig. 4b), mitochondrial ultrastructure (Supplementary Fig. 4e), or mitochondrial reactive oxygen species (ROS) levels (Supplementary Fig. 5a,b). In addition, SNRK overexpression or knockdown in HL1 cardiac cells led to no change in mitochondrial content (Supplementary Fig. 4c), mitochondrial morphology (Supplementary Fig. 4d) or mitochondrial ROS levels (Supplementary Fig. 5c,d). SNRK TG mice also had no change in expression of a panel of antioxidant genes (Supplementary Fig. 5e), and superoxide dismutase 2 (SOD2) protein levels were unchanged in SNRK TG mice (Supplementary Fig. 5f) and in HL1 cells with SNRK overexpression (Supplementary Fig. 5g). These data indicate that SNRK does not induce significant mitochondrial structural differences or ROS production in the heart. Together, these studies suggest that mitochondria from SNRK TG hearts are more efficient and exhibit decreased uncoupling.

### SNRK decreases metabolic flux through PPARα and UCP3

**Figure 3:**
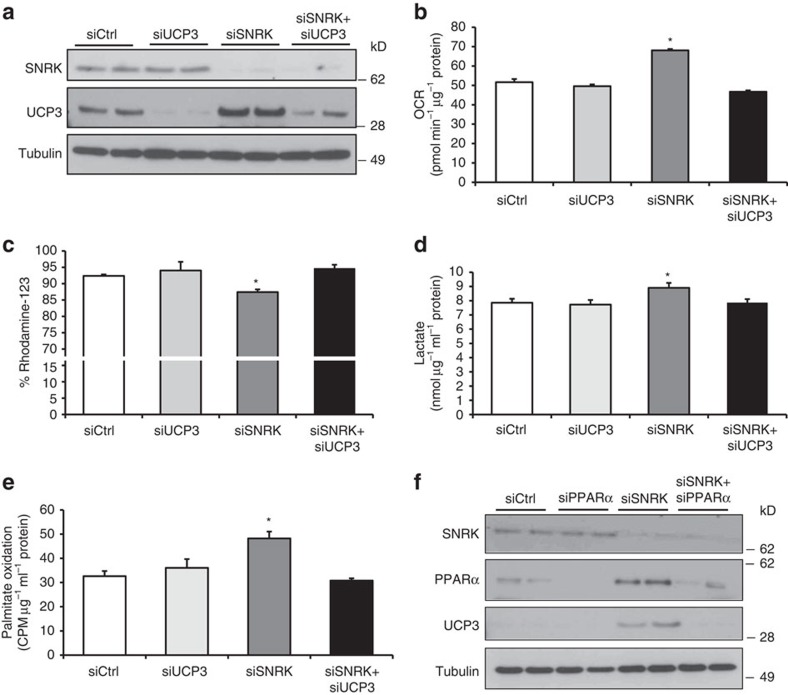
SNRK decreases mitochondrial efficiency through PPARα and UCP3. (**a**) Protein levels of SNRK, UCP3, and tubulin in HL1 cells with SNRK and UCP3 knockdown. siCtrl indicates scrambled control siRNA, siUCP3 indicates UCP3 siRNA, siSNRK indicates SNRK siRNA, and siSNRK+siUCP3 indicates treatment with both siRNAs. Oxygen consumption rate (OCR, **b**, *n*=6), mitochondrial membrane potential (**c**, *n*=3), lactate levels (**d**, *n*=6), and palmitate oxidation (**e**, *n*=3) in HL1 cells with SNRK and/or UCP3 knockdown. (**f**) Protein levels of SNRK, PPARα, UCP3, and tubulin in HL1 cells with SNRK and/or PPARα knockdown. Labels above blots indicate siRNAs used. Experiment is representative of three independent samples. Data are represented as mean±s.e.m. *P≤0.05 by one-way ANOVA. ANOVA, analysis of variance.

To investigate the mechanism of decreased mitochondrial uncoupling by SNRK, we studied knockdown of endogenous SNRK in HL1 cardiomyocytes. Knockdown of SNRK resulted in increased levels of UCP3 (Fig. 3a), increased oxygen consumption (Fig. 3b), decreased mitochondrial membrane potential (Fig. 3c), increased lactate levels (Fig. 3d) and increased fatty acid oxidation (Fig. 3e). All of these changes were reversed with UCP3 knockdown (Fig. 3b–e), demonstrating that UCP3 mediates mitochondrial coupling and metabolic substrate usage by SNRK. Additionally, overexpression of SNRK led to a decrease in oxygen consumption, which was reversed with combined overexpression of UCP3 (Supplementary Fig. 3a). UCP3 mRNA levels were also suppressed by SNRK overexpression (Supplementary Fig. 3b).

Since PPARα levels were decreased in the hearts of SNRK TG mice and PPARα is known to regulate UCP3 (ref. 17), we next explored the role of PPARα in mediating the changes of UCP3 by SNRK. We first demonstrated that SNRK knockdown upregulates PPARα, and that PPARα knockdown can reverse the effect of SNRK knockdown on UCP3 levels (Fig. 3f). We also used a construct containing the UCP3 promoter driving the expression of firefly luciferase^18^, and showed that SNRK knockdown induces the UCP3 promoter, while a mutant construct with deletion of a PPARα-responsive element did not cause any change in luciferase activity (Supplementary Fig. 3e). The effect of SNRK knockdown on the UCP3 promoter was reversed with PPARα knockdown. These results suggest that SNRK regulates the transcription of UCP3 through PPARα.

### SNRK decreases PPARα and UCP3 through Trib3 upregulation

**Figure 4:**
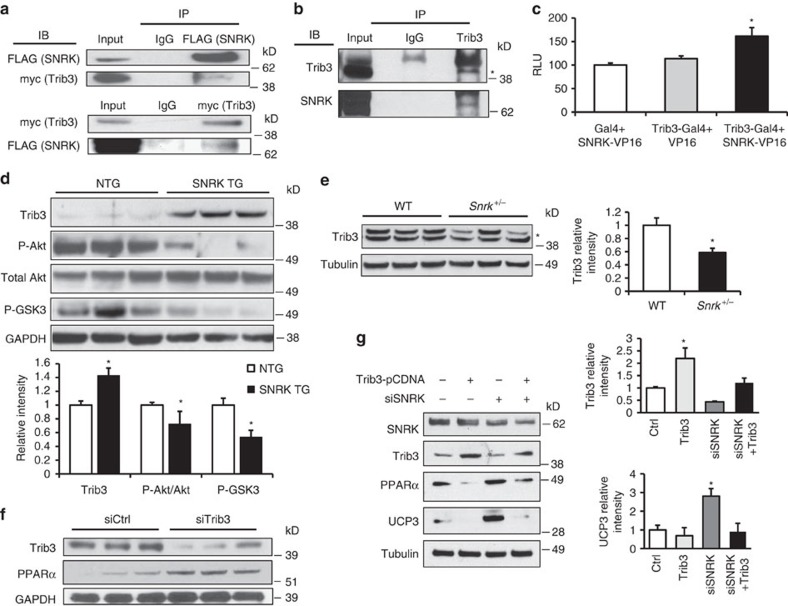
SNRK decreases PPARα and UCP3 through upregulation of Trib3. (**a**) Co-immunoprecipitation of FLAG-SNRK and myc-Trib3 in HEK293 cells. IP: immunoprecipitation antibody, IB: immunoblot antibody. (**b**) Co-immunoprecipitation of endogenous SNRK with Trib3 from mouse hearts. Asterisk indicates Trib3 band. (**c**) Mammalian two hybrid assay of SNRK-VP16 and Trib3-GAL4 in HEK293 cells. RLU, relative luminescence units. *n*=9. (**d**) Western blots of Trib3, p-S473 Akt, total Akt, p-GSK3 and GAPDH in NTG and SNRK TG hearts. Graphs show summary of *n*=8 for Trib3 relative to loading control, *n*=8–9 for p-Akt relative to total Akt, and *n*=4 for p-GSK3 relative to GAPDH. (**e**) Western blots of Trib3 and tubulin in WT and *Snrk*^+/−^ hearts. Asterisk denotes nonspecific band. Densitometry shows summary of *n*=4–5. (**f**) Western blots of Trib3, PPARα, and GAPDH in HL1 cells treated with control or Trib3 siRNA. (**g**) Western blots of SNRK, Trib3, PPARα, UCP3 and tubulin in HL1 cells transfected with pCDNA or Trib3-pCDNA and control or SNRK siRNA. Densitometry shows summary of *n*=5. Data are represented as mean±s.e.m. *P≤0.05 by one-way ANOVA or Student’s *t*-test. ANOVA, analysis of variance.

Next, we investigated the mechanism of regulation of PPARα by SNRK. SNRK has no known protein substrates, so we conducted a yeast two hybrid screen using SNRK as bait in order to identify novel binding partners of SNRK. Tribbles homologue 3 (Trib3), a known inhibitor of Akt signalling^19^, emerged as a positive hit from this screen. We verified that Trib3 binds to SNRK in mammalian cells through co-immunoprecipitation of overexpressed Trib3 and SNRK with pulldown of either protein (Fig. 4a). In addition, we were able to detect pulldown of endogenous SNRK with Trib3 in mouse hearts (Fig. 4b), and confirmed the pulldown results by showing increased interaction of SNRK and Trib3 in a mammalian two hybrid assay in HEK293 cells (Fig. 4c). SNRK TG mice had increased expression of Trib3, as well as decreased phosphorylation of Akt and its substrate GSK3 (Fig. 4d). Conversely, *Snrk*^+/−^ mice had decreased protein levels of Trib3 (Fig. 4e). Neither SNRK TG nor *Snrk*^+/−^ mice had altered levels of Trib3 mRNA expression (Supplementary Fig. 3c,d). Overexpression of the wild-type kinase-active SNRK, but not the kinase-inactive SNRK-T173A mutant^7^,^11^, increased stability of Trib3 protein after cycloheximide treatment (Supplementary Fig. 6a). Moreover, knockdown of SNRK destabilized Trib3 expression (Supplementary Fig. 6b), suggesting that SNRK activity increases Trib3 protein stability.

Previously, PPARα was reported to induce Trib3 expression in the liver^20^, and Trib3 increases proteasomal degradation of PPARα in skeletal muscle^21^, but it is not known whether and how Trib3 regulates PPARα and UCP3 in the heart. We demonstrated that Trib3 knockdown leads to increased PPARα in cardiac cells (Fig. 4f), and SNRK knockdown destabilized Trib3 protein (Supplementary Fig. 6b) and stabilized PPARα protein expression after cycloheximide treatment (Supplementary Fig. 6c), suggesting that SNRK may regulate PPARα and UCP3 levels through stabilized Trib3. To study this hypothesis, we overexpressed Trib3 in combination with SNRK knockdown in cardiac cells. SNRK knockdown alone reduced Trib3 and increased PPARα and UCP3 expression (Fig. 4g). In the presence of Trib3 overexpression, SNRK knockdown no longer induced PPARα and UCP3, indicating that SNRK regulates PPARα and UCP3 through Trib3. Moreover, this regulation occurs partially through a transcriptional mechanism, as Trib3 overexpression suppressed expression from a luciferase construct driven by the promoter of PPARα (Supplementary Fig. 3f).

### SNRK protects against ischaemia-reperfusion (I/R) injury

**Figure 5:**
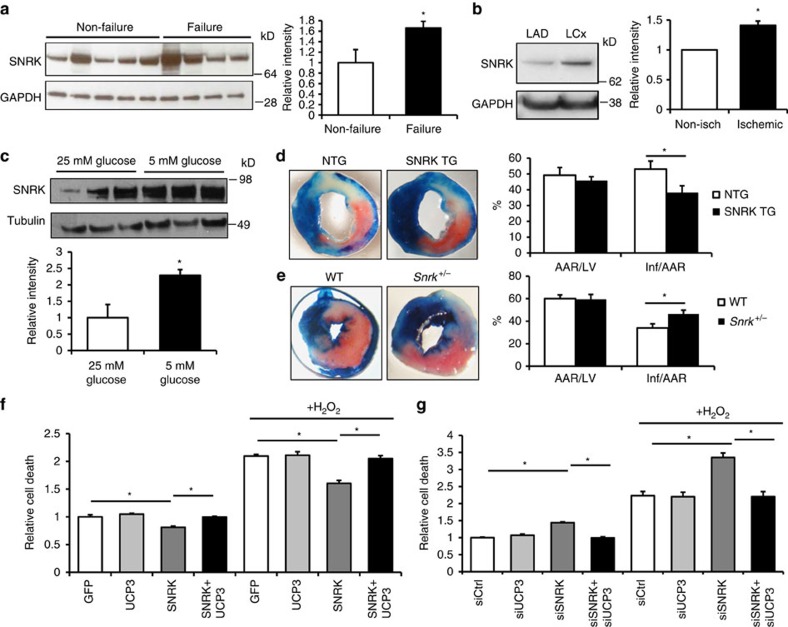
SNRK decreases tissue damage in response to ischemia/reperfusion. (**a**) SNRK protein levels in human hearts from patients with no apparent cardiac disease (non-failure hearts) and from patients with cardiomyopathy (failure hearts). (**b**) SNRK protein levels in dog hearts in the non-ischemic left anterior descending artery (LAD) and in the ischemic left circumflex artery (LCx). Densitometry shows summary of three independent samples. (**c**) SNRK protein levels in HL1 cells grown for 36 h in 5 or 25 mM glucose-containing media. (**d**) Representative images of hearts from NTG and SNRK TG mice subjected to I/R. Graph shows summary of six mice for each group. (**e**) Representative images of hearts from WT and *Snrk*^+/−^ mice subjected to I/R. Graphs show summary of four WT mice and seven *Snrk*^+/−^ mice. (**f**) Cell death in HL1 cells with overexpression of GFP, SNRK and/or UCP3 after hypoxia, without and with H_2_O_2_ treatment. (**g**) Cell death in HL1 cells with control, SNRK, and/or UCP3 siRNA treatment after hypoxia treatment, without and with H_2_O_2_ treatment. Data are represented as mean±s.e.m. *P≤0.05 by Student’s *t*-test or one-way ANOVA. ANOVA, analysis of variance.

We next hypothesized that SNRK may reduce cardiac damage that occurs in response to low oxygen and substrate availability during ischaemia, since SNRK overexpression leads to improved mitochondrial efficiency. First, we found that SNRK protein levels are increased in hearts from patients with cardiomyopathy relative to patients with normal cardiac function (Fig. 5a). SNRK protein was also increased in ischaemic regions of dog hearts subjected to myocardial infarction, compared with non-ischaemic control regions (Fig. 5b). These findings demonstrate that SNRK protein is upregulated in response to cardiac ischaemia, and suggest that SNRK may play an adaptive role during prolonged ischaemia. Upregulation of SNRK could also be recapitulated in HL1 cells treated with low-glucose media (Fig. 5c), demonstrating that SNRK levels are increased by lower metabolic substrate availability.

We next investigated whether SNRK confers changes in metabolism under the conditions of hypoxic and hydrogen peroxide stress. Under normoxia and hypoxia-reoxygenation with and without hydrogen peroxide in HL1 cells, SNRK overexpression decreased oxygen consumption (Supplementary Fig. 7a), palmitate oxidation (Supplementary Fig. 7b) and lactate production (Supplementary Fig. 7c), but did not affect ATP production (Supplementary Fig. 7d) relative to the empty vector control. The kinase-inactive SNRK-T173A mutant did not significantly decrease these parameters relative to the control (Supplementary Fig. 7a–d). These findings indicate that SNRK regulates metabolism and decreases oxygen consumption in the setting of hypoxia-reoxygenation, and suggest that SNRK may be protective during ischaemia/reperfusion.

We then studied whether increased SNRK expression in SNRK TG mice is protective against tissue damage caused by acute I/R. SNRK TG mice had reduced infarct size compared with NTG controls (Fig. 5d) following I/R, as demonstrated by decreased tissue death evidenced by perfused Evans Blue staining. Conversely, loss of SNRK in the *Snrk*^+/−^ mice led to an increase in infarct size compared with WT controls (Fig. 5e). Overall these results indicate that, consistent with its role in inducing mitochondrial efficiency, SNRK protects against ischaemic cardiac damage and tissue death.

Lastly, we investigated whether the protection against cell death by SNRK was mediated by mitochondrial uncoupling. SNRK overexpression decreased cell death in response to hypoxia both without and with H_2_O_2_ in HL1 cells (Fig. 5f). However, this protection was reversed when UCP3 was overexpressed with SNRK. Conversely, SNRK downregulation increased cell death, with concomitant downregulation of UCP3 decreasing cell death to control levels (Fig. 5g). Together these data demonstrate that SNRK decreases cell death in a UCP3-dependent manner, suggesting that the effects of SNRK on mitochondrial coupling are needed for protection against cell death, although other mechanisms may also contribute.

## Discussion

Our results characterize a mouse model of cardiac mitochondrial efficiency, in which overexpression of SNRK decreases metabolic substrate usage and oxygen consumption but maintains cardiac function and energy, while a reduction in SNRK levels has the opposite effect. This model demonstrates both decreased substrate flux and enhanced mitochondrial coupling. We also identify a binding partner of SNRK, Trib3, which is upregulated by SNRK in mice and mediates the regulation of PPARα and UCP3 by SNRK. Since UCP3 knockdown reverses the effects of SNRK knockdown on metabolic flux, our results also suggest that mitochondrial coupling, at least partially, contributes to a decrease in demand for metabolic substrate and a subsequent decrease in glucose and fatty acid consumption. Together, our work suggests that SNRK regulates cardiac mitochondrial efficiency and substrate flux through Trib3, PPARα and UCP3, and that SNRK is protective during ischaemia.

Basal proton leak has been found to contribute to up to 12% of oxygen consumption in the heart^22^,^23^, with mitochondrial proton leak increasing significantly following I/R injury^24^. The reduction in basal cardiac proton leak in the SNRK TG mice, as reflected by basal oxygen consumption rates, is consistent with these previous findings (Fig. 1f). SNRK TG mice were also protected against I/R injury, a state during which mitochondrial proton leak increases substantially.

Since SNRK decreases UCP3 expression, alternative consequences of this downregulation should be considered. In addition to uncoupling, UCP3 plays a role in mitochondrial fatty acid export and ROS dissipation^25^,^26^. We have found no change in ROS levels with SNRK manipulation, and SNRK protects against I/R injury and cell death, suggesting that there is no significant increase in ROS levels under the conditions that we have tested. In addition, because we have found no increase in ROS levels by SNRK, there are likely adaptive or compensatory mechanisms at play that are regulated by SNRK and that counteract the ROS that could be potentially generated by downregulation of UCP3. Moreover, previous studies regarding UCP3 in cell death were performed in KO mouse models of UCP3 (refs 27, 28), whereas SNRK TG mice have loss of but not complete ablation of UCP3. Additional studies on other signalling pathways regulated by SNRK, and the effects of different levels of UCP3 expression on cell death, will help to reveal precisely how SNRK decreases cell death.

In addition, as shown by our data, UCP3 knockdown or overexpression alone is not sufficient to cause changes in substrate usage or cell death. Indeed, the SNRK TG mice do not phenocopy the UCP3 KO or PPARα KO mice^29^,^30^, and the relationship of UCP3 to mitochondrial efficiency and cell death is complex^29^,^31^. We have determined that the context of increased SNRK expression and/or activity is necessary for UCP3 to enable cardiac protection, suggesting that SNRK likely mediates metabolic effects through additional pathways, even though it is dependent on UCP3 to increase mitochondrial efficiency and substrate flux. Investigation into other pathways regulated by SNRK will reveal precisely what signalling mechanisms may differ between SNRK and UCP3 mouse models. Additional *in vivo* models with both SNRK and UCP3 manipulation in the setting of ischaemia/reperfusion will also be useful in this regard.

We have identified Trib3 as a novel binding partner of SNRK that is upregulated in SNRK TG mice, and the consequences of this upregulation should also be considered. As Trib3 is an inhibitor of Akt, and Akt can act as a pro-survival kinase^32^, it may be expected that some increases in cell death may occur with increased Trib3 expression. We did not observe any increase in tissue death in response to I/R; instead, we saw that the SNRK TG mice are more resistant to this insult. These results suggest that the increase in Trib3 by SNRK does not cause a sufficient deficit in Akt activity to cause cell death, as SNRK also exerts protective effects by improving mitochondrial efficiency. In addition, LKB1 KO in skeletal muscle was previously found to increase Trib3 levels^33^. As we have found that SNRK increases Trib3 levels in the heart, the effects of the LKB1 KO model are presumably affecting other downstream kinases or affecting tissue-specific pathways that causes differential effects on Trib3 expression.

Since the SNRK TG mice have decreased substrate oxidation with maintained cardiac function, and SNRK was found to be increased in chronic ischaemic conditions, the TG mice could be stated to be merely adapting to the change in SNRK levels. However, our additional data demonstrating that SNRK increases mitochondrial coupling and is protective against cell death in a UCP3-dependent fashion suggests that SNRK specifically regulates substrate oxidation and coupling, which would not occur with a simple adaptive response. However, though our data suggest that UCP3 and effects on metabolic flux are needed to decrease oxygen demand, they do not preclude that additional pathways may also contribute by decreasing ATP demand in the hearts, which could include changes in calcium handling efficiency, ionic gradient maintenance, and myofibrillar contractility.

In summary, our study demonstrates that SNRK is a regulator of cardiac energetics with functional effects that are unique from other kinases. SNRK decreases oxygen consumption and metabolic flux through increased Trib3 expression and subsequent PPARα-dependent UCP3 downregulation. SNRK also enables the maintenance of cardiac energy levels and function, and improves the response to myocardial I/R. These findings identify SNRK as a potential target for modifying cardiac mitochondrial efficiency.

## Methods

### Generation of transgenic and knockout mice

A transgene containing full-length human SNRK with an eGFP tag at its C-terminus was cloned downstream of the α–MHC promoter (a kind gift of Dr Jeffrey Robbins, Cincinnati Children’s Hospital). A fragment containing the promoter and transgene was agarose gel-purified and used in a microinjection of the pronucleus of one-cell mouse embryos of B6SJL mice at the Northwestern University Transgenic and Targeted Mutagenesis Facility. Two founders of transgenic mice were identified by traditional PCR of genomic DNA (primer sequences 5′-TTTCTCTGACCACATGGCAG/TTGAAGTCGATGCCCTTCAG-3′), real-time PCR of total cardiac DNA and RNA, and Western blotting of total cardiac protein. One line with a twofold increase in SNRK expression relative to endogenous expression was selected for further study. Transgenic mice were backcrossed at least five generations to C57BL/6 wild-type mice before experiments. The SNRK KO-first promoter-driven construct was obtained from Knockout Mouse Project Repository. C57BL/6-derived embryonic stem cells containing the SNRK KO construct with a neomycin cassette before exon 3 were injected into the pronucleus of one-cell mouse embryos of C57BL/6 mice to generate the *Snrk*^+/−^ mice. Floxed SNRK mice were generated by mating the *Snrk*^+/−^ mice with Flp recombinase mice to remove the neomycin cassette. Homozygous floxed *Snrk* mice were mated to α-MHC-Cre mice to generate the cardiac-specific KO mice. KO was confirmed by PCR, PCR with reverse transcription and western blotting. All mice were compared only to nontransgenic or wild-type gender-matched littermates (both males and females were included), and were between 6 and 20 weeks old.

### Animal studies

Animal studies were approved by the Institutional Animal Care and Use Committee of Northwestern University and the University of Utah. Animals were housed with 12 h dark and light cycles with free access to traditional chow and water. Before harvesting hearts or other tissue, mice were deeply anaesthetized with avertin and killed by cervical dislocation. For isolated working heart experiments, mice were anaesthetised with chloral hydrate.

### Materials and reagents

HEK293 cells were obtained from ATCC, and HL1 cells were purchased from Sigma-Aldrich. siRNA for SNRK, UCP3, PPARα and Trib3 were purchased from Dharmacon. Plasmids used in cell culture transfection experiments using Lipofectamine 2000 or Lipofectamine LTX were SNRK-pEGFP-N3, SNRK-T173A-pEGFP-N3, UCP3-pEGFP-N3 and Trib3-pCDNA3.

### Western blotting and quantitative real-time PCR

Tissue lysates were prepared in a Triton X-100 buffer (50 mM Tris-HCl pH 7.5, 1 mM EGTA, 1 mM EDTA, 1% v/v Triton X-100, 0.27 M sucrose, and a phosphatase inhibitor cocktail consisting of 1 mM sodium orthovanadate, 10 mM sodium beta-glycerophosphate, 50 mM sodium fluoride, 5 mM sodium pyrophosphate) for analysis of SNRK, an IGEPAL buffer (20 mM Tris-HCl pH 7.5, 3 mM EDTA, 3 mM EDTA, 125 mM NaCl, 0.25% IGEPAL, phosphatase inhibitor cocktail) for analysis of Trib3, Akt and GSK3α/β, and RIPA buffer for PPARα and UCP3, with all buffers containing protease inhibitors. Protein concentration was measured by Bradford reagent or the BCA method, as appropriate. Equal protein concentration was loaded into a precast NuPAGE SDS–polyacrylamide gel electrophoresis gel and transferred to nitrocellulose for blotting. SNRK antibody was from Millipore (#07-720, 1:1,000 working concentration) or Sigma (#HPA042163, 1:500), GFP antibody was from Santa Cruz (#sc-9996, 1:1,000), P-Akt Ser473 (#9271, 1:1,000), Akt (#9272, 1:1,000), P-GSK3α/β Ser21/9 (#8566, 1:1,000), total AMPK (#2532, 1:1,000) and P-AMPKα Thr172 (#2531, 1:1,000) antibodies were from Cell Signaling, UCP3 antibody was from Abcam (#ab3477, 1:1,000), PPARα antibody was from Abcam (#ab8934, 1:3,000), and Trib3 antibody was from Millipore (#07-2160, 1:3,000). Unedited western blots are shown in Supplementary Fig. 8.

Total RNA was isolated from mouse hearts using the RNA-Stat 60 reagent (Tel-test), followed by DNAse treatment and RNA precipitation by ethanol. Quantitative real-time PCR was conducted using SYBR Green dye, with primer specificity determined by dissociation curve analysis. Primer sequences were as follows: UCP3 5′-GGGACCCACGGCCTTCTA/CAAACATCATCACGTTCCAAGCT-3′, PPARα—5′-ACAACCCGCCTTTTGTCATA/CTCGGCCATACACAAGGTCT-3′, Cox1—5′-AGTGCTAGCCGCAGGCATTACTAT/CTGGGTGCCCAAAGAATCAGAACA-3′, Trib3—5′-GCCTACGTGGGACCAGAGATAC/CTCCAGACATCAGCCGCTTT-3′, 18s—5′-GTAACCCGTTGAACCCCATT/CCATCCAATCGGTAGTAGCG-3′.

### SNRK kinase activity assay

SNRK activity was determined in total mouse heart lysates from NTG and SNRK mice, and in anti-GFP immunoprecipitates from total lysates. Heart homogenates were obtained by dounce homogenization in Triton X-100 lysis buffer containing no EDTA. For total heart lysates, equal protein content was incubated with 100 μM ATP and 10 mM MgAc, with or without H3.3 substrate, for one hour at room temperature. Activity was assessed by measurement of ATP using the ADP-Glo Kinase Assay (Promega), and activity with H3.3 added was normalized to activity without H3.3.

### Echocardiography

Cardiac function was determined non-invasively by echocardiography^34^ using the Vevo 770 high-resolution imaging system with a 30 MHz scan head. Mice were anaesthetised using isoflurane via nasal cone, the chest was shaved and the temperature of the mice was maintained at 37 °C. The heart rate was continually monitored. Ultrasound gel was applied, and the scan head was used to obtain long- and short-axis views. B-mode guided M-mode echocardiography was performed to assess cardiac function. At least ten independent cycles were obtained per experiment.

### Perfused working hearts

Glycolysis, glucose oxidation, and palmitate oxidation were measured in isolated perfused working hearts from 16-week-old gender-matched SNRK TG and NTG littermates^35^. The hearts were perfused with KHB buffer gassed with 95% O_2_, 5% CO_2_ and supplemented with 0.4 mM palmitate bound to 3% bovine serum albumin (BSA) and 5 mM glucose. Glucose and palmitate metabolic rates were measured over a 60 min period by the release of ^3^H_2_O or ^14^CO_2_ from [5-^3^H]glucose and [U-^14^C]glucose, or [9,10-^3^H]palmitate. Metabolic rates were normalized to dry heart weight and rate-developed product pressure (heart rate multiplied by left ventricular developed pressure). Cardiac power was calculated as the product of cardiac output multiplied by left ventricular developed pressure.

For *Snrk*^+/−^ and cs*Snrk* KO experiments, 15–16-week-old gender-matched littermates were perfused with 5 mM glucose and 1.0 mM palmitate bound to 3% BSA. An increased level of palmitate was used to enhance its contribution to overall ATP production so that an increase in palmitate metabolism could be more readily observed. TG and KO studies were performed independently by investigators at different sites.

### ATP determination

ATP was determined from flash-frozen working heart samples by high-performance liquid chromatography at the HPLC Analytical Core at the University of Alberta.

### Mitochondrial function

Mitochondrial oxygen consumption was measured in mitochondria isolated from mouse hearts using the Seahorse XF24 Extracellular Flux Analyzer^36^. Mouse hearts were minced in cold fibre relaxation buffer (100 mM KCl, 5 mM EGTA, 5 mM HEPES pH 7.0) and homogenized in HES buffer (5 mM HEPES, 1 mM EDTA, 0.25 M sucrose, pH 7.4) using a glass dounce homogenizer. The homogenate was centrifuged twice at 500 *g*, then the supernatant was centrifuged at 9,000 *g* to obtain a crude mitochondrial pellet which was resuspended in HES buffer with 0.2% free fatty acid-free BSA. Equal protein amounts of mitochondria were diluted in 450 μl total volume of MAS buffer (70 mM sucrose, 220 mM mannitol, 5 mM KH_2_PO_4,_ 5 mM MgCl_2_ 5 mM, 2 mM HEPES, 1 mM EGTA, 0.2% fatty acid-free BSA, pH 7.4) and were loaded into an XF24 plate with centrifugation at 2,000 *g*. Following centrifugation, MAS buffer containing 10 mM pyruvate and malate were added and the plate was incubated for 8 min at 37 °C without CO_2_. Oxygen tension was measured in the Seahorse XF24 Extracellular Flux Analyzer following injections of 250 μM ADP, 2 μM oligomycin, 4 μM carbonyl cyanide 3-chlorophenylhydrazone (CCCP), and 4 μM antimycin.

### Neonatal mouse cardiomyocytes and TMRE staining

Neonatal mouse cardiomyocytes were obtained from 1- to 3-day-old SNRK TG or NTG control mouse hearts following euthanization by cervical dislocation^37^. The ventricles were removed, washed in HBSS lacking Ca^2+^ and Mg^2+^, and minced. The cells were dissociated at 37 °C in 5% CO_2_ for 15 min in HBSS lacking Ca^2+^ and Mg^2+^ and containing 0.25% trypsin. The cells were centrifuged and resuspended in fetal bovine serum-minimum essential media (FBS-MEM), then pre-plated for 2 h. The suspended cells were plated on cover slips and used for experiments. Tetramethylrhodamine ethyl ester (TMRE) fluorescence was measured in a flow-through chamber mounted on an inverted fluorescent microscope^38^. The cells were loaded for 1 h with 100 nM TMRE in a humidified incubator, then perfused for 1 h in a flow-through chamber with buffered salt solution containing 10 nM TMRE to establish baseline Δψm. Digital images were obtained every 1–3 min using an oil immersion lens. Fluorescence intensity for cells and background regions were analysed. The cells were then perfused with oligomycin (2.5 μg ml^−1^) to increase Δψm to the maximum, and then CCCP (4 μM) to diffuse Δψm to the minimum. Baseline fluorescence was compared with minimum CCCP fluorescence to determine the percentage of mitochondrial coupling.

### Metabolic assays in HL1 cardiomyocytes

HL1 cells were cultured in Claycomb media (Sigma-Aldrich) containing 10% serum, 0.1 mM norepinephrine, 2 mM L-Glutamine, and penicillin/streptomycin. Knockdown in HL1 cardiomyocytes was accomplished using Dharmafect reagent in media containing L-glutamine only. Cells were treated with equal amounts of total siRNA. For transfection experiments, HL1 cells were treated with siRNA, and 24 h later transfected with 1:3 DNA:Lipofectamine LTX with PLUS reagent and used in experiments 48 h later.

Oxygen consumption was performed in the Seahorse Bioscience XF24 Bioanalyzer. 60,000 cells were seeded per well, and 24 h later were treated with siRNA. Forty-eight hours later, cells were used for oxygen consumption studies using unbuffered DMEM+1 mM sodium pyruvate+1 g l^−1^ glucose, pH 7.4, using the manufacturer’s protocol. Oxygen consumption readings were normalized to total protein per well.

For Rhodamine-123 measurement, siRNA-treated cells were changed to serum-free Claycomb media+L-glutamine, trypsinized, resuspended in 30 nM Rhodamine-123 (Life Technologies) for 30 min at room temperature, and then analysed by flow cytometry.

Lactate levels were measured in six-well plates of siRNA-treated cells using the Colorimetric/Fluorometic Lactate Assay Kit (Biovision). Readings from media and cells combined were normalized to protein content.

Fatty acid oxidation was performed by measuring ^3^H_2_O generated from [9,10-^3^H]-palmitic acid (Perkin-Elmer). Cells were incubated in serum-free Claycomb media containing 0.2 mM sodium palmitate-BSA with trace amounts of radiolabelled palmitic acid for four hours. Media was removed from the cells and 10% trichloroacetic acid was added for 60 min at 4 °C. The protein was pelleted, and NaOH was added to the supernatant to a final concentration of 1 N. The supernatant was run through a 0.2 g ml^−1^ Dowex 1 × 2–400 column (Sigma-Aldrich), then eluted with H_2_O into scintillation vials. Readings from media alone were subtracted as background, and the subtracted readings were normalized to protein content of cells.

### Yeast two hybrid screens

Yeast two hybrid screens were performed at the Indiana University Yeast Two Hybrid Facility. Full-length human SNRK protein was used as bait and cloned into the pGBKT7 vector containing a GAL4 DNA-binding domain. The screen was performed against a mouse embryonic fibroblast cDNA library fused to a GAL4 activation domain (Clontech, Mountain View, CA, USA), with screening conducted on more than 10 × 10^6^ clones. Clones passing both prototrophic (growth on His-dropout media with 20 or 100 mM of 3AT, or growth on Ura-dropout media) and lacZ growth tests were selected for sequencing.

### Co-immunoprecipitation and mammalian two hybrid assay

Co-immunoprecipitation in HEK293 cells was performed by cloning a FLAG tag at the N-terminus of full-length human SNRK and a myc tag at the N-terminus of full-length human Trib3, with both genes cloned into the pCMVScript vector. HEK293 cells (cultured at 37 °C and 5% CO_2_ in Cell Gro MEM media with 1 mM sodium pyruvate, 10% FBS, and penicillin/streptomycin) were co-transfected with each construct for 48 h using Lipofectamine 2000, then collected in Triton-X buffer. Protein G Sepharose beads were pre-conjugated to either anti-FLAG or anti-mouse IgG, or anti-myc or anti-rabbit IgG antibodies. The cell lysate was pre-cleared on Protein G Sepharose for 30 min, then added to the antibody-conjugated beads for one hour at 4 °C. The beads were washed three times for 20 min using lysis buffer. LDS Sample buffer and Reducing agent (Invitrogen) were added directly to the beads for 10 min at 70 °C, and the supernatant was loaded onto a precast NuPAGE SDS–polyacrylamide gel electrophoresis gel for Western blotting.

For endogenous co-immunoprecipitation, heart tissue was homogenized in IP buffer containing 25 mM Tris pH 7.5, 150 mM NaCl, 1 mM EDTA, 5% glycerol and 1% NP40 supplemented with protease and phosphatase inhibitors. 1 mg protein was diluted in IP buffer containing 0.1% NP40 and pre-cleared with Protein G Agarose beads (Roche). The pre-cleared lysates were incubated with 2 ug of Trib3 antibody (Calbiochem) overnight, and the beads were washed in IP buffer containing 0.1% NP 40 and added to the lysate for 7 h. The beads were washed four times with IP buffer containing 0.1% NP40 and boiled in 4 × LDS sample loading buffer containing sample reducing reagent before loading the gel.

Mammalian Two Hybrid Assay was conducted using the Checkmate Mammalian Two Hybrid System from Promega according to the company protocol. Briefly, HEK293 cells were co-transfected with SNRK-VP16 and GAL4 plasmids, GAL4-Trib3 and VP16 plasmids, or SNRK-VP16 and Trib3-GAL4 plasmids. These plasmids were co-transfected with the pG5luc luciferase vector. A positive protein:protein interaction was indicated by firefly luciferase expression, which was measured in cell lysates with Dual Glo Luciferase reagent (Promega), and normalized to renilla expression.

### Human cardiac ischaemia samples

Non-failing and failing human heart samples were obtained from Northwestern Memorial Hospital. The failing ischaemic human heart tissues were procured from the explanted hearts of cardiac transplant recipients. Non-failing samples were obtained from unmatched organ donors with no history of cardiac disease whose EFs were >55% and whose hearts were unsuitable for transplantation. The explanted hearts were immediately placed in cold cardioplegic solution, then flash-frozen in liquid nitrogen. Frozen samples were homogenized in lysis buffer (1% Nonidet P-40, 10% glycerol, 137 mM NaCl, 20 mM Tris-Cl, pH 7.4, 1 mM phenylmethylsulfonyl fluoride, 20 mM NaF, 1 mM sodium pyrophosphate, 1 mM sodium orthovanadate and 2 μg ml^−1^ each of aprotinin and leupeptin), centrifuged at 37,500 *g* for 25 min at 4 °C, and used in western blotting^39^. Protocols for tissue procurement were approved by the Institutional Review Board of Northwestern Memorial Hospital. Informed consent was obtained from all transplant patients and organ donor families before tissue collection.

### Canine ischaemia tissue samples

Dog tissue samples were kindly provided by Dr Robert Decker (Northwestern University). Experimental ischaemic animal preparation was performed on dogs following an overnight fast and 3 weeks of on-site conditioning, with all procedures approved by the Institutional Animal Care and Use Committee of Northwestern University^40^. Dogs were subjected to 75% left circumflex coronary artery occlusion under continuous hemodynamic monitoring for 5 h, then killed with pentobarbital and potassium chloride. Cardiac tissue samples were homogenized on ice in radioimmune precipitation assay buffer with the addition of protease inhibitors (Roche Applied Science), and 40 μg of protein was used for western blotting^39^.

### Ischaemia/reperfusion and histological analysis

TG and KO I/R studies were performed independently by different investigators. Mice were anaesthetized with 3% isoflurane, put in the supine position, and monitored with ECG leads^41^. Body temperature was monitored by rectal probe and maintained at 37 °C with heating pads. Mice were ventilated by a catheter placed in the trachea and connected to a mouse ventilator. Ventilation was maintained at a tidal volume of 200 μl at a rate of 105 breaths/minute. The chest was opened by incision of the left fourth intercostal space. A 1 mM piece of PE-10 tubing was placed on the left anterior descending artery, and a knot was placed on the tube with an 8-0 silk suture to occlude the coronary artery. Ischaemia was verified by pallor of the left ventricular anterior wall and ST segment evaluation and QRS widening on the ECG. After 40 min of occlusion, the suture on the PE-10 tubing was cut to allow for reperfusion. The chest was then closed in layers. The mice were kept on heating pads, ventilated on 100% oxygen by nasal cannula, and given buprenorphine for post-operative pain.

Forty-eight hours after I/R, mice were anaesthetized with an intraperitoneal injection of avertin. The animals were respirated with a ventilator. The suture was re-occluded, and 500 μl of 5% Evans Blue was injected into the right ventricle. Hearts were excised and sectioned transversely into five sections, and then incubated in 2% triphenyltetrazolium chloride (TTC, Sigma-Aldrich) for 10 min at 37 °C, followed by 10% neutral-buffered formaldehyde for 24 h.

Sections were weighed and photographed using a Leica microscope, then analysed using Image J (National Institutes of Health). Viable myocardium stained red, and the infarcted areas appeared pale. No-risk area was determined by Evans Blue staining.

The size of infarction was determined by the following equations: Weight of infarction=(A1 × W1)+(A2 × W2)+(A3 × W3)+(A4 × W4)+(A5 × W5), where A is per cent area of infarction by planimetry and W is the weight of each section; Percentage of infarcted left ventricle=(weight of infarction/weight of LV) × 100; AAR as a percentage of LV=(weight of LV—weight of LV stained blue)/weight of LV.

### Cell death

HL1 cells were subjected to hypoxia for 16 h and reoxygenation for 2 h. H_2_O_2_ was added at 500 μM where indicated after 6 h of hypoxia. Cells are then washed with Hank's balanced salt solution (HBSS) and stained with propidium iodide (Sigma) and Hoechst 33342 (Life Technologies). Images were taken on an epifluorescnece microscope, and cell death was quantified as the percentage of cells with PI staining over total number of cells.

### Study approval

All animal studies were reviewed and approved by the Northwestern Animal Care and Use Committee, Chicago, IL, USA. Non-failing human hearts were obtained from donor cadavers and were therefore exempted from IRB review. Failing human heart samples were obtained from the cardiac transplanted patients at Northwestern University. Tissue samples were obtained from patients consenting to tissue collection in the Cardiac Surgery Outcomes Registry (IRB# STU00012288), which was approved by the Northwestern University Institutional Review Board (IRB).

### SNRK kinase activity using anti-GFP immunoprecipitates

For activity with anti-GFP immunoprecipitates, 750 μg of lysate was pre-cleared on Protein G Sepharose beads, and 1 μl of anti-GFP (Abcam #ab290) was conjugated to 100 μl of Protein G Sepharose beads for 1 h. The pre-cleared lysates were added to the conjugated beads for 1 h at 4 °C, then the beads were washed three times in lysis buffer. The beads were incubated with or without H3.3 substrate for 1 h at room temperature with 100 μM ATP and 10 mM MgAc in lysis buffer, followed by assessment of kinase activity in the supernatant and normalization of signal with H3.3 added to signal without H3.3.

### ATP production assay

HL1 cells were washed with incubation buffer (0.25 M sorbitol, 2 mM KH_2_PO_4_, 5 mM MgCl_2_, 1 mM EDTA, 0.1% fatty acid-free BSA, 20 mM MOPS, pH 7.4) and permeabilized in incubation buffer with 10 μg ml^−1^ digitonin. After permeabilization, cells were incubated in incubation buffer with ADP (1 mM), with or without malate (5 mM) and pyruvate (5 mM), or succinate (5 mM) for 15 min at 37°. ATP levels were determined with the ATP Determination Kit (Invitrogen) according to the manufacturer’s instructions and normalized to protein content of the same well.

### Triglyceride and glycogen determination

Triglyceride content was measured in equal weights of flash-frozen heart tissue. Tissue was homogenized in 2:1 chloroform:methanol, 0.2 volume of methanol was added, and the samples were spun for 10 min at 3,500 r.p.m. A volume of 0.04% CaCl_2_ equal to 0.2 of the total volume was added to the supernatant, the mixture was spun for 20 min at 2,400 r.p.m., and the supernatant was washed three times with 3% Chloroform/48% Methanol. 50 μl of methanol was added, then the mixture was dried overnight. The pellet was resuspended in 3:2 tert-butyl alcohol:triton X-100/methanol, incubated overnight, and triglyceride levels were measured using the Triglyceride L-type TG M Kit (Wako Diagnostics).

Glycogen levels were measured in flash-frozen heart tissue using the Glycogen Determination Kit (Biovision) and readings were normalized to protein content.

### Mitochondrial complex activity

Mitochondrial complex activity was determined in mouse heart homogenates using the MitoSciences Mitochondrial Complex I, II, and IV Activity Assays according to the manufacturer’s protocol.

### Mitochondrial DNA content

Mitochondrial DNA content was measured from total DNA isolated from mouse hearts using the Qiagen DNeasy Kit. Quantitative real-time PCR using SYBR Green and primers for Cytochrome c oxidase 1 (Cox1) and 18S rRNA was performed to measure mitochondrial and nuclear DNA, respectively.

### Mitotracker green

Cells were stained with 200 nM MitoTracker Green FM (Life Technologies) for 15 min, and the nuclei were counterstained with Hoechst 34322 (Life Technologies). Images were captured using a Zeiss epifluorescence microscope.

### Electron microscopy

For electron microscopy studies, left ventricle cardiac tissue was fixed in cold 2.5% glutaraldehyde and 2.0% paraformaldehyde in 0.1 M sodium cacodylate buffer, treated with 2% osmium tetroxide in 0.1 M sodium cacodylate, en-bloc stained in 3% aqueous uranyl acetate, dehydrated in ethanol, and embedded in epoxy-araldite resin^42^. 70 nm sections were cut with a Leuica UC6 ultramicrotome and examined with an FEI Tecnai Spirit transmission electron microscope. Mitochondrial size was analysed using ImageJ.

### MitoSox red

MitoSox Red (Invitrogen) was used to assess mitochondrial O_2_· production. 5 mM MitoSox (Life Technologies) was added to the cells in the presence or absence of 100 ug ml^−1^ Antimycin A (Sigma) to maximize ROS production from mitochondria. Cells were visualized by microscopy and ROS levels were quantified by ImageJ software. Four fields per each sample were obtained and averaged. Nuclei were counterstained with Hoescht and subtracted from the total Mitosox fluorescence to exclude the signal from nonspecific localization of dye into the nucleus.

For mitochondrial studies, isolated mitochondria were resuspended in HES buffer (5 mM HEPES, 1 mM EDTA, 0.25 M sucrose, pH 7.4 adjusted with 1 M KOH) with 0.2% fatty acid-free BSA. Mitosox was added to the mitochondrial suspension in the presence or absence of Antimycin A (Sigma). The mitochondria were stained for 15 min. The fluorescent intensity was analysed on a BD Canto flow cytometer.

### Luciferase reporter assays

For the UCP3 luciferase reporter assay, mouse embryonic fibroblasts treated with nonsilencing control, mouse SNRK siRNA and/or PPARα siRNA were transfected with 200 ng of the firefly reporter construct containing the full-length mouse UCP3 promoter (from position −2,063 to +63) or a deletion mutant of the UCP3 promoter lacking a PPARα-responsive element (with the last 250 base pairs deleted, both kind gifts from Dr Michael Sack at the National Institutes of Health) and 50 ng of Renilla luciferase construct using Lipofectamine (Invitrogen) according to the manufacturer’s instructions. Twenty-four hours after transfection, the cells were lysed in 1 × passive lysis buffer (Promega). The lysates were loaded onto a 96-well plate, and the luciferase activity was determined using the Dual Luciferase Assay System (Promega) with a Modulus Microplate Reader (Turner Biosystems). The ratio between firefly and renilla luciferase activity was normalized to that of empty vector under different treatments.

For the PPARα promoter reporter assay, mouse embryonic fibroblasts treated with control or Trib3 siRNA were transfected with a PPARα promoter construct^43^ (a kind gift from Dr Daniel Kelly at The Scripps Institute). The luciferase assay was conducted as above.

### Statistical analysis

Data are presented as mean±s.e.m. Statistical analysis was conducted using Student’s *t*-test for comparison of two groups, or analysis of variance analysis for three groups followed by *posthoc* analysis using Tukey’s honest significant difference (HSD) or Fisher’s least significant difference (LSD) test. A *P* value of less than or equal to 0.05 was considered significant.

### Data availability

The authors declare that relevant data supporting the conclusions of this paper are contained within the article and the Supplementary Information, or can be made available upon reasonable request.

## Additional information

**How to cite this article:** Rines, A. K. *et al*. Snf1-related kinase improves cardiac mitochondrial efficiency and decreases mitochondrial uncoupling. *Nat. Commun.* **8**, 14095 doi: 10.1038/ncomms14095 (2017).

**Publisher's note:** Springer Nature remains neutral with regard to jurisdictional claims in published maps and institutional affiliations.

## Supplementary Material

Supplementary Information Supplementary Figures and Supplementary Tables.
