# Corrigendum: Snf1-related kinase improves cardiac mitochondrial efficiency and decreases mitochondrial uncoupling

**DOI:** 10.1038/ncomms16155

**Published:** 2017-08-30

**Authors:** Amy K. Rines, Hsiang-Chun Chang, Rongxue Wu, Tatsuya Sato, Arineh Khechaduri, Hidemichi Kouzu, Jason Shapiro, Meng Shang, Michael A. Burke, Eltyeb Abdelwahid, Xinghang Jiang, Chunlei Chen, Tenley A. Rawlings, Gary D. Lopaschuk, Paul T. Schumacker, E. Dale Abel, Hossein Ardehali

Nature Communications 8: Article number: 14095; DOI: 10.1038/ncomms14095 (2017); Published: 01 24 2017; Updated: 08 30 2017.

The authors inadvertently omitted Eltyeb Abdelwahid, who contributed to the generation of animal models and their initial evaluation, from the author list. This has now been corrected in both the PDF and HTML versions of the Article.

