# Supplementary material for: Snf1-related kinase improves cardiac mitochondrial efficiency and decreases mitochondrial uncoupling
Source: Nat Commun. 2017 Jan 24;8:14095. doi: 10.1038/ncomms14095 (PMC5286102; doi:10.1038/ncomms14095)
Supplement: Supplementary Information — Supplementary Figures and Supplementary Tables. [file ncomms14095-s1.pdf]

Supplementary Figure 1

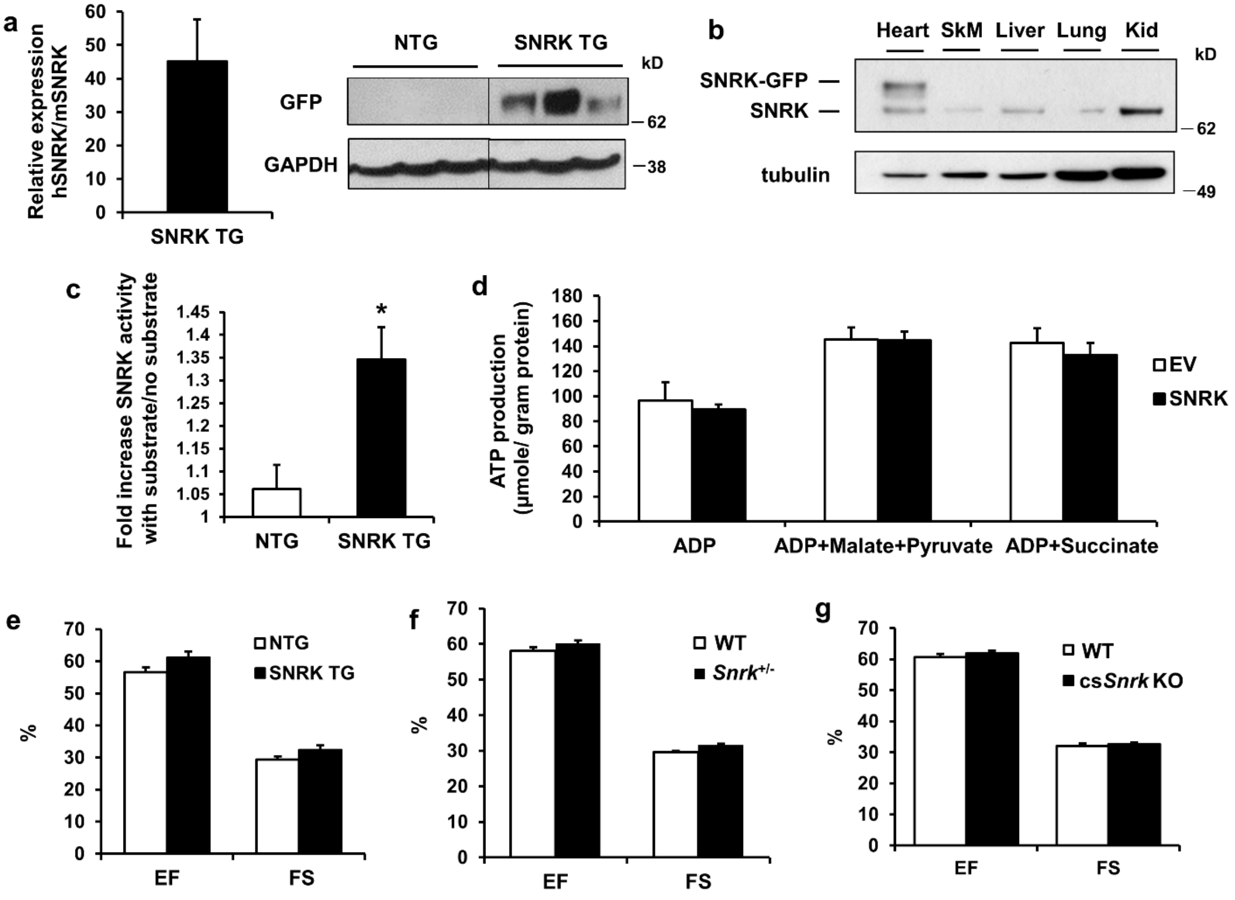

Supplementary Figure 1: SNRK expression and activity in SNRK TG mice, and cardiac performance in SNRK TG, *Snrk*$^{+/-}$, and cs*Snrk* KO mice. (**a**) mRNA expression of the human SNRK transgene relative to the endogenous mouse SNRK in SNRK TG mice. n=5. Western blot shows SNRK-GFP expression in SNRK TG hearts with GFP antibody. (**b**) Protein expression of endogenous SNRK, transgenic SNRK-GFP (with SNRK antibody), and tubulin in the heart, skeletal muscle (SkM), liver, lung, and kidney (Kid) in a SNRK TG mouse. (**c**) SNRK activity as measured in anti-GFP immunoprecipitates in NTG and SNRK TG hearts with H3.3 substrate and normalized to background signal without H3.3 substrate. n=4. (**d**) ATP production in HL1 cardiomyocytes with empty vector (EV) or SNRK overexpression. ATP was measured in the presence of substrates indicated. n=16. (**e**) Cardiac ejection fraction (EF) and fractional shortening (FS) in NTG and SNRK TG mice. n=16-18. (**f**) Cardiac EF and FS in WT and *Snrk*$^{+/-}$ mice. n=3. (**g**) Cardiac EF and FS in WT and cs*Snrk* KO mice. Data are represented as mean ± SEM. **P*≤0.05 by Student's t-test.

Supplementary Figure 2

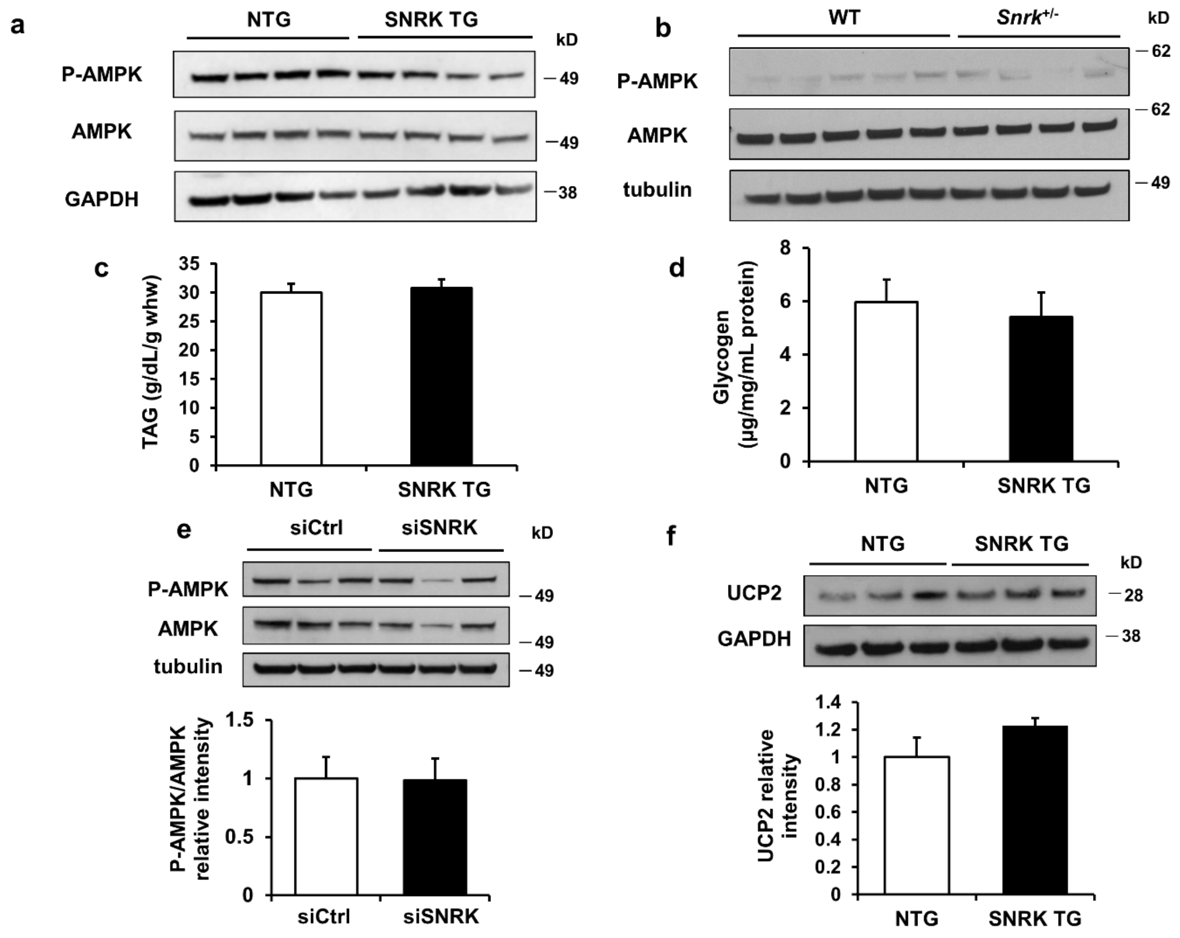

Supplementary Figure 2: SNRK TG mice do not have increased levels of P-AMPK or UCP2, nor depleted triglyceride or glycogen stores. (**a**) P-AMPK protein levels in NTG and SNRK TG hearts. (**b**) P-AMPK protein levels in WT and *Snrk*$^{+/-}$ hearts. Triglyceride (TAG, **c**) and glycogen content (**d**) in NTG and SNRK TG hearts. n=6. (**e**) P-AMPK protein levels in HL1 cells with control or SNRK siRNA treatment. (**f**) UCP2 protein levels in NTG and SNRK TG hearts. Data are represented as mean ± SEM.

Supplementary Figure 3

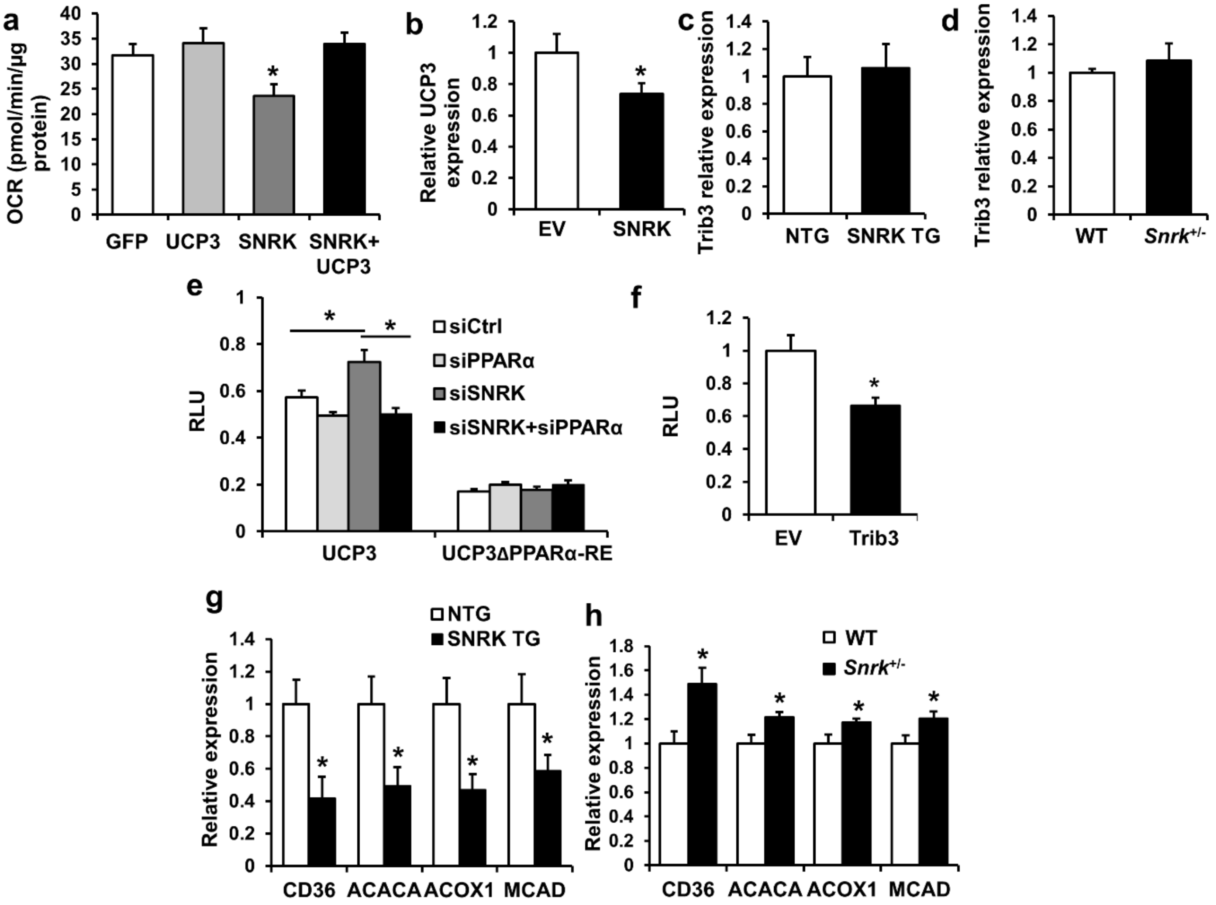

Supplementary Figure 3: SNRK decreases oxygen consumption in a UCP3-dependent manner and increases expression of the UCP3 promoter through PPARα. (**a**) Oxygen consumption in HL1 cells with overexpression of GFP, UCP3, SNRK, or SNRK+UCP3. n=3. (**b**) UCP3 mRNA levels with SNRK overexpression in HL1 cells. n=3. Trib3 mRNA levels in (**c**) NTG and SNRK TG hearts, n=4-5, and (**d**) WT and $SNRK^{+/-}$ hearts, n=3. (**e**) Luciferase activity from a UCP3 promoter-luciferase construct and a construct with a PPARα-response element deleted (UCP3ΔPPARα-RE) with SNRK and/or PPARα knockdown in mouse embryonic fibroblasts. n=3. (**f**) Luciferase activity from a PPARα promoter-luciferase construct with overexpression of an empty vector (EV) or Trib3. n=3. (**g**) PPARα target gene expression in NTG and SNRK TG hearts. n≥3. (**h**) PPARα target gene expression in WT and *Snrk*$^{+/-}$ hearts. n≥3. Data are represented as mean ± SEM. *P≤0.05 by one-way ANOVA or Student's t-test.

Supplementary Figure 4

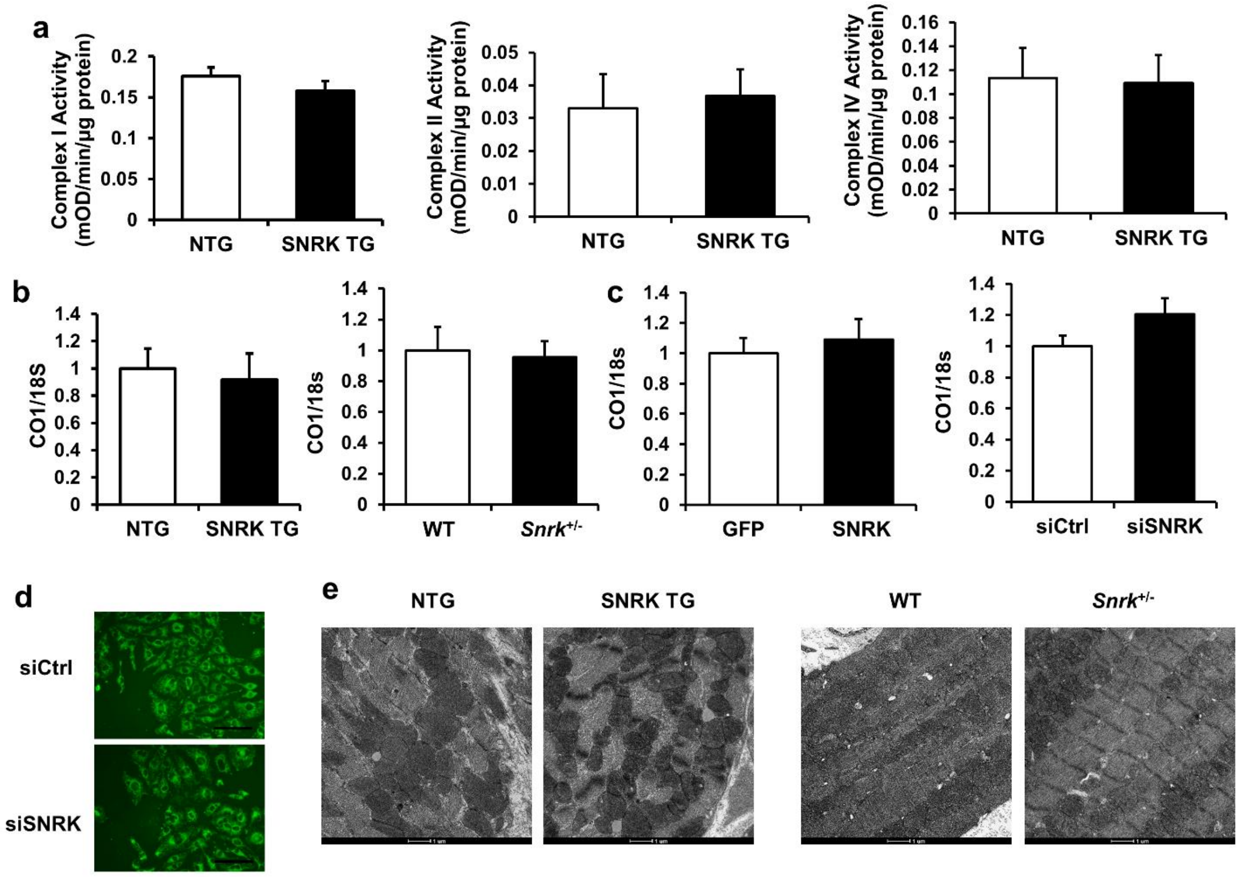

Supplementary Figure 4: Mitochondrial complex activity, content, and ultrastructure are not altered by SNRK. (**a**) Mitochondrial complex I, II, and IV activity in NTG and SNRK TG hearts. n=6. (**b**) Mitochondrial DNA content in NTG and SNRK TG mouse hearts (n=6), and WT and *Snrk*$^{+/-}$ hearts (n=3). (**c**) Mitochondrial DNA content in HL1 cells with GFP or SNRK overexpression, or control or SNRK siRNA treatment. n=3. (**d**) Representative MitoTracker Green images of mitochondria morphology in HL1 cells with control or SNRK siRNA treatment. Scale bar, 100 µm. (**e**) Representative electron microscopy images of mitochondrial ultrastructure in NTG and SNRK TG hearts, and in WT and SNRK$^{+/-}$ hearts. Scale bar, 1 µm. Data are represented as mean ± SEM.

Supplementary Figure 5

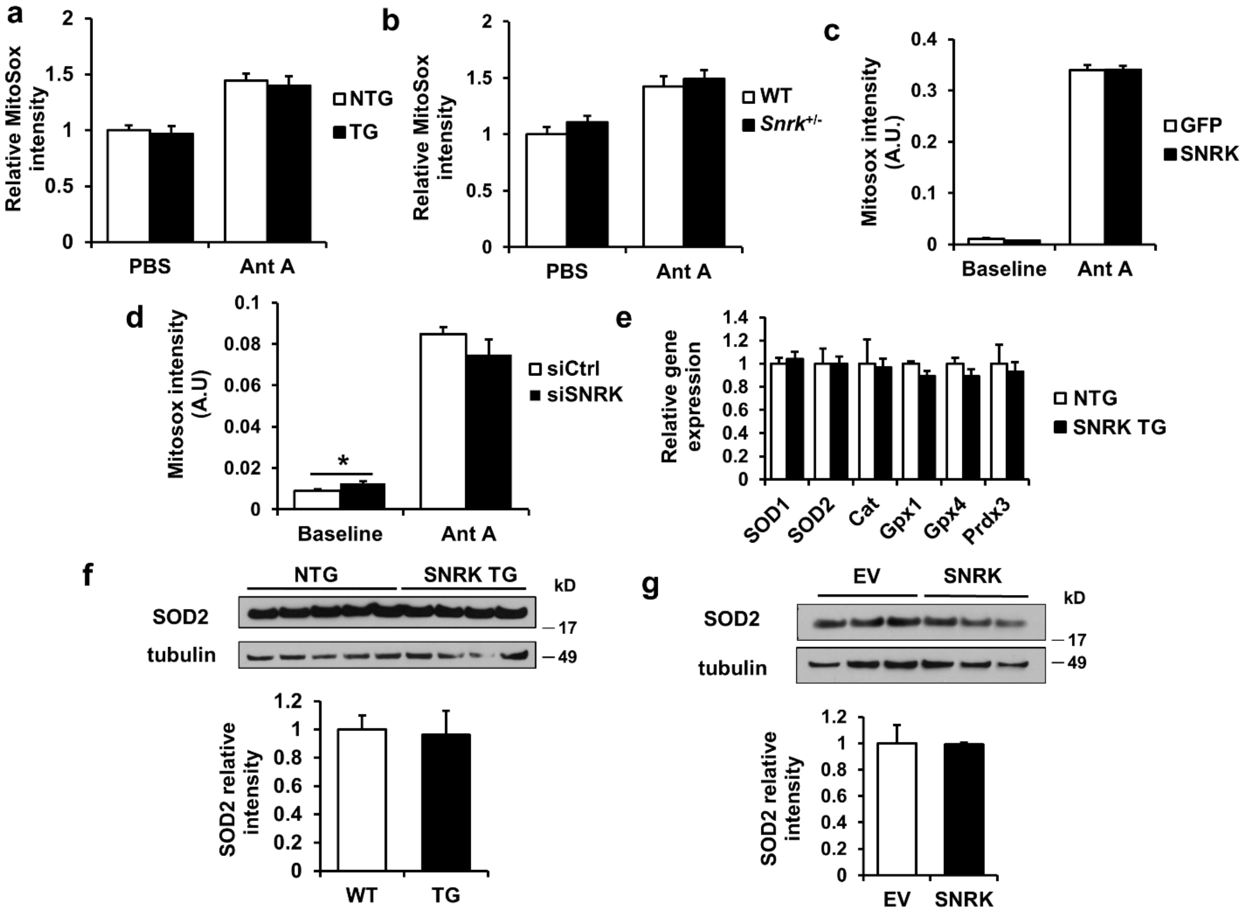

Supplementary Figure 5: Mitochondrial ROS are not altered by SNRK. (**a**) Mitochondrial ROS in isolated mitochondria from NTG or SNRK TG hearts as measured by Mitosox intensity at baseline and following treatment of mitochondria with Antimycin A (Ant A). n=3. (**b**) Mitochondrial ROS in isolated mitochondria from WT or *Snrk*$^{+/-}$ hearts. n=3. (**c**) Mitochondrial ROS in HL1 cells with GFP or SNRK overexpression. n=3. (**d**) Mitochondrial ROS in HL1 cells with control (siCtrl) or SNRK siRNA treatment. n=3. (**e**) Antioxidant gene expression in NTG and SNRK TG hearts. SOD = superoxide dismutase, Cat = catalase, Gpx = glutathione peroxidase, Prdx = peroxiredoxin. n=3. (**f**) SOD2 expression in NTG and SNRK TG hearts. (**g**) SOD2 expression in HL1 cardiomyocytes with empty vector (EV) or SNRK overexpression. Data are represented as mean ± SEM. *$P \leq 0.05$ by Student's t-test.

Supplementary Figure 6

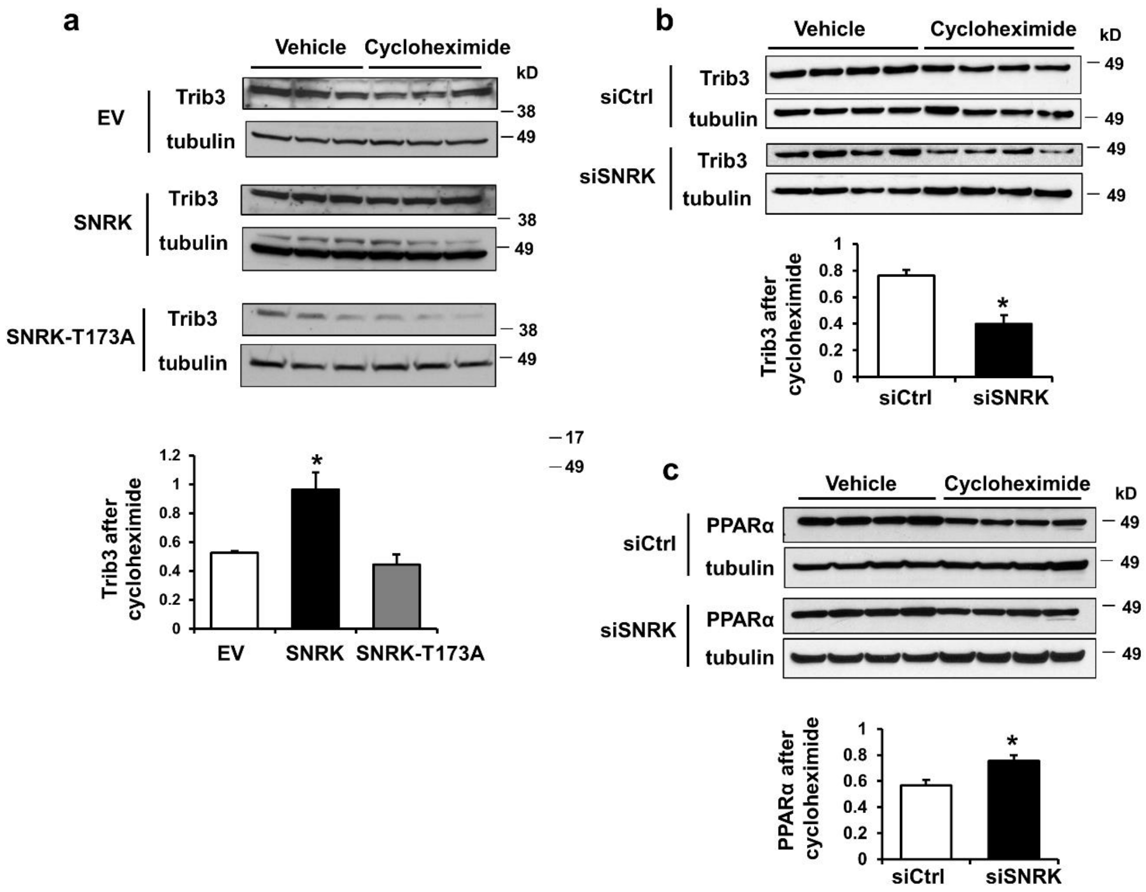

Supplementary Figure 6: SNRK increases protein stability of Trib3 and decreases stability of PPARα. (**a**) Trib3 protein levels in HL1 cardiomyocytes with empty vector, SNRK, or SNRK-T173A overexpression without and with 4 hours of 150 µg/mL cycloheximide treatment. (**b**) Trib3 protein levels in HL1 cardiomyocytes with control or SNRK siRNA treatment without and with cycloheximide treatment. (**c**) PPARα protein levels in HL1 cardiomyocytes with control or SNRK siRNA treatment without and with cycloheximide treatment. Data are represented as mean ± SEM. **P*≤0.05 by one-way ANOVA or Student's t-test.

Supplementary Figure 7

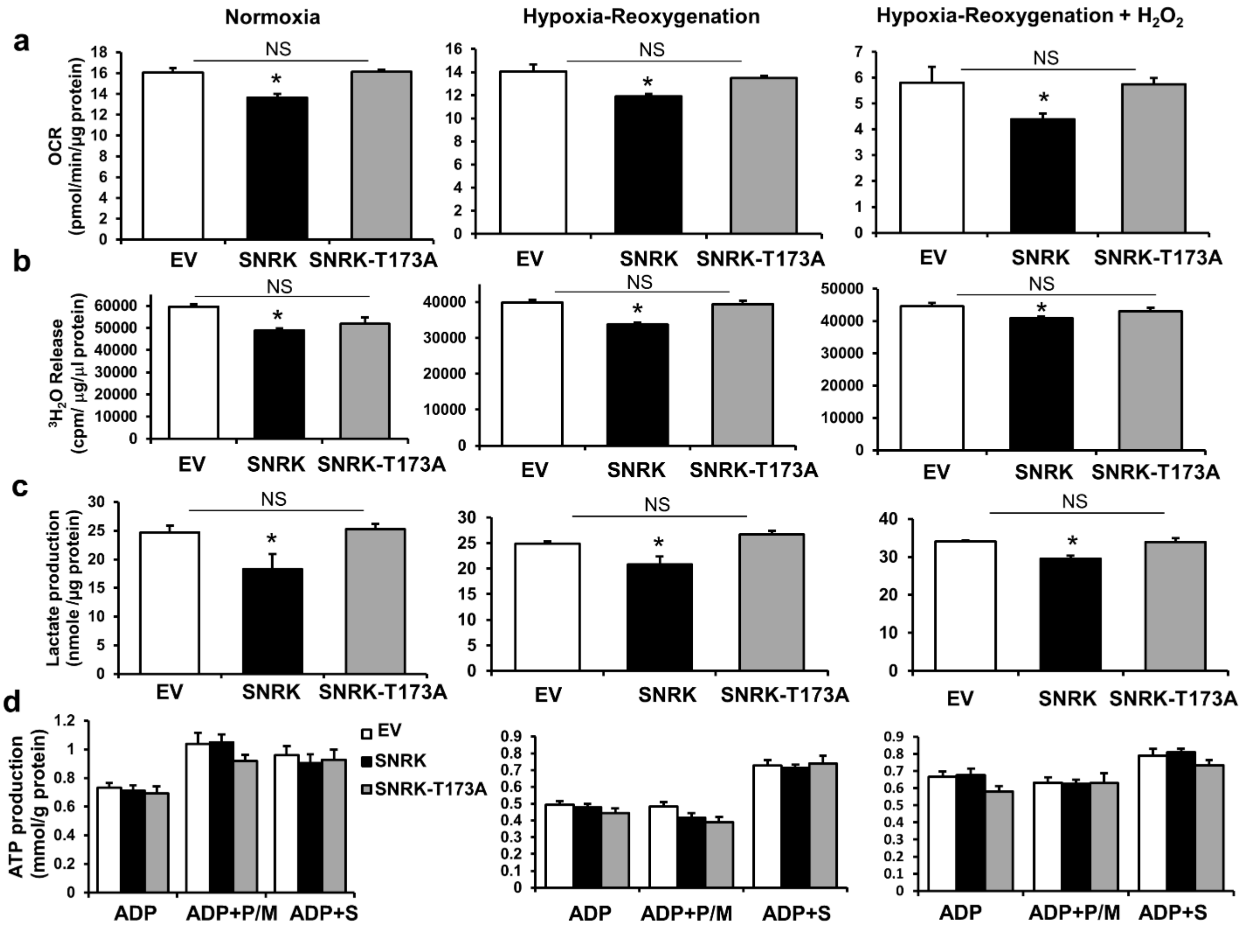

Supplementary Figure 7: SNRK decreases oxygen consumption and metabolic flux during hypoxia-reoxygenation. (**a**) Oxygen consumption in HL1 cells with empty vector (EV) control, SNRK, or SNRK-T173A overexpression in the presence of normoxia (n=29-32), hypoxia-reoxygenation (n=14-16), or hypoxia-reoxygenation with H_2O_2 treatment (n=14-16). (**b**) Palmitate oxidation in HL1 cells with control, SNRK, or SNRK-T173A overexpression in the presence of normoxia, hypoxia-reoxygenation, or hypoxia-reoxygenation with H_2O_2 treatment. n=3-5. (**c**) Lactate production in HL1 cells with control, SNRK, or SNRK-T173A overexpression in the presence of normoxia (n=3), hypoxia-reoxygenation (n=4-6), or hypoxia-reoxygenation with H_2O_2 treatment (n=3-4). (**d**) ATP production in HL1 cells with control, SNRK, or SNRK-T173A overexpression in the presence of normoxia, hypoxia-reoxygenation, or hypoxia-reoxygenation with H_2O_2 treatment. n=11-14. P/M = pyruvate/malate, S = succinate. n≥3. Data are represented as mean ± SEM. *$P \leq 0.05$ by Student's t-test.

Supplementary Figure 8

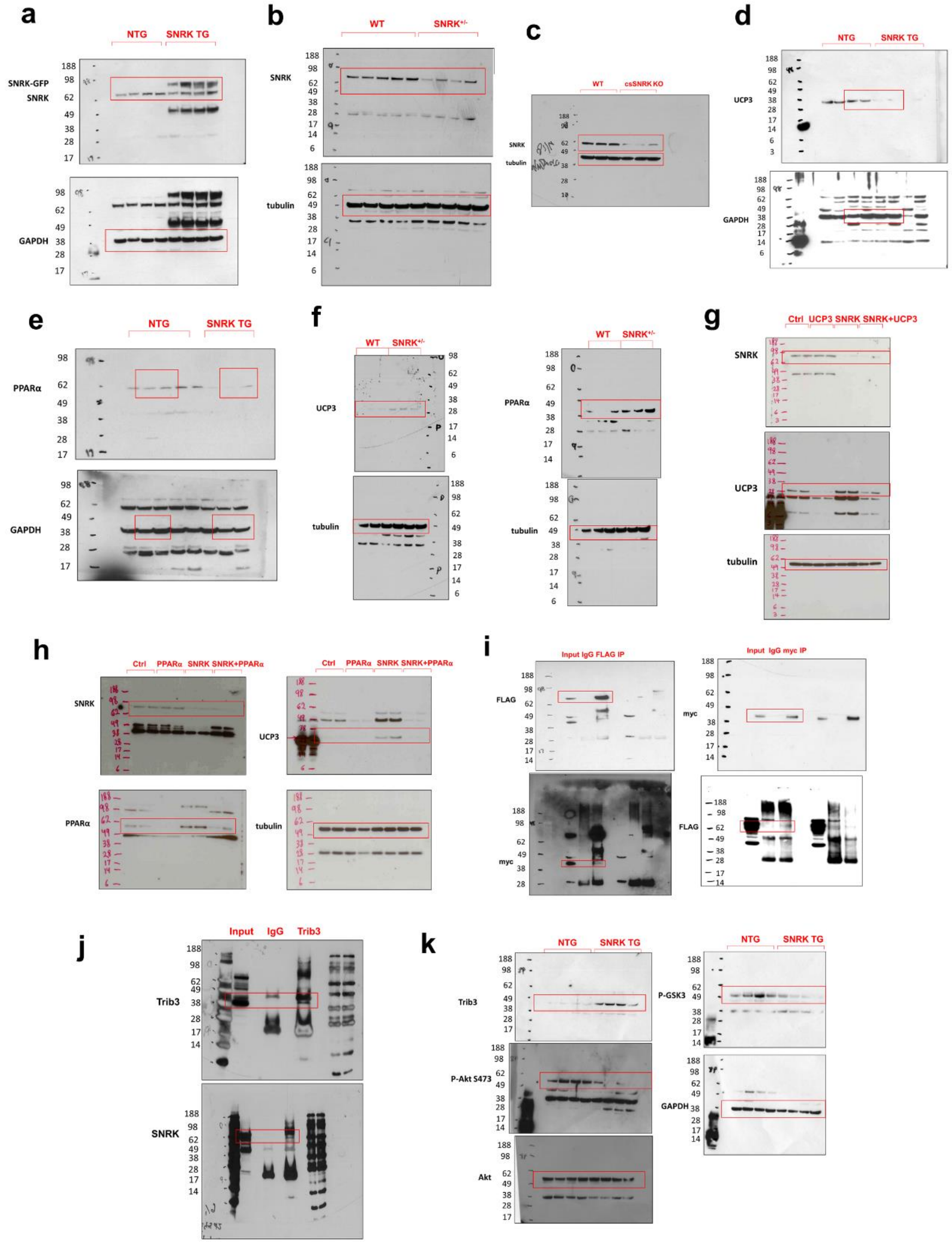

Supplementary Figure 8: Unedited Western blots. Uncut blots for (**a**) Fig. 1a, (**b**) Fig. 1g, (**c**) Fig. 1j, (**d**) Fig. 2e, (**e**) Fig. 2e, (**f**) Fig. 2g, (**g**) Fig. 3a, (**h**) Fig. 3f, (**i**) Fig. 4a, (**j**) Fig. 4b, and (**k**) Fig. 4d.

Supplementary Figure 8

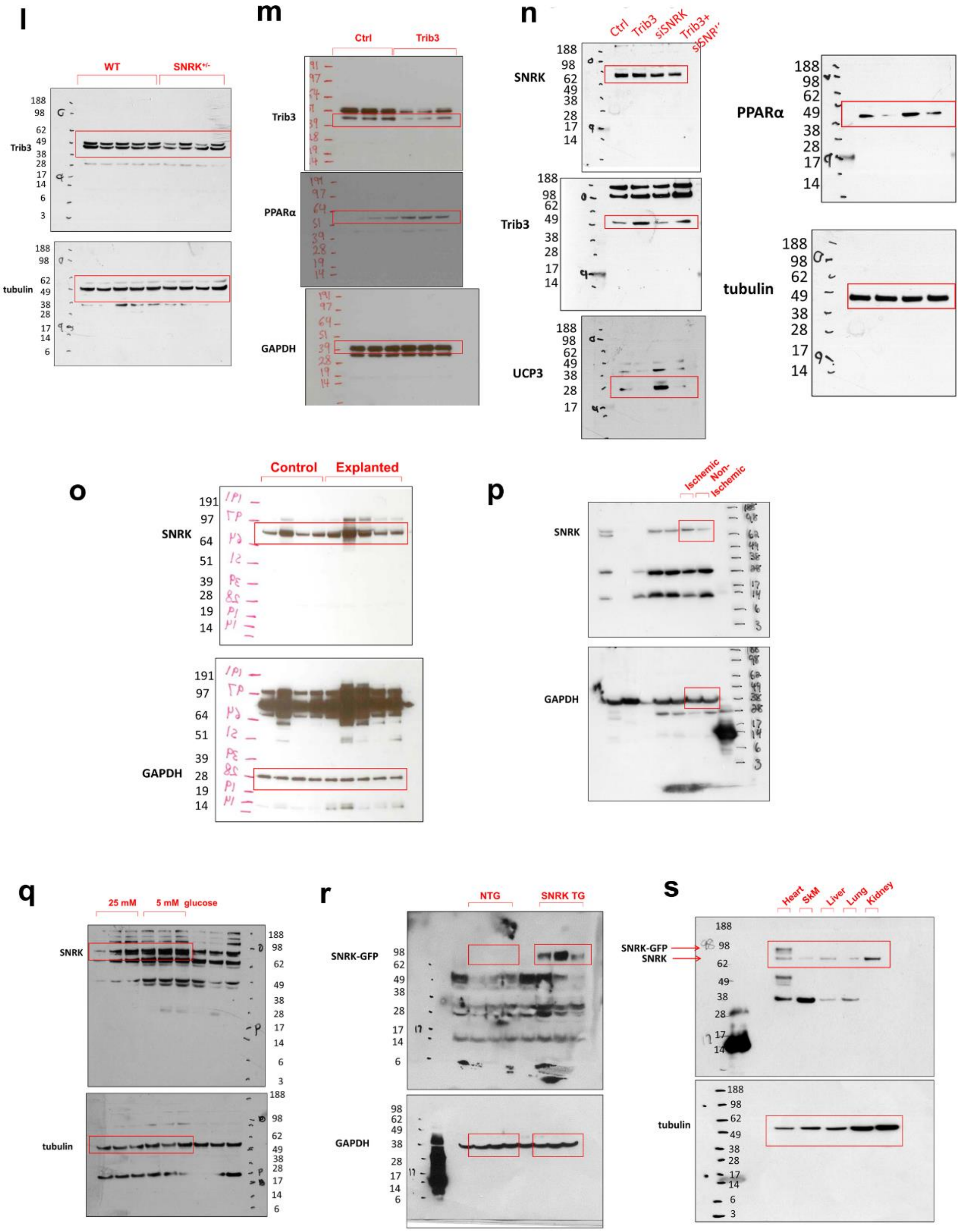

Supplementary Figure 8: Unedited Western blots. Uncut blots for (**l**) Fig. 4e, (**m**) Fig. 4f, (**n**) Fig. 4g, (**o**) Fig. 5a, (**p**) Fig. 5b, (**q**) Fig. 5c, (**r**) Supplemental Fig. 1a, and (**s**) Supplemental Fig. 1b.

Supplementary Figure 8

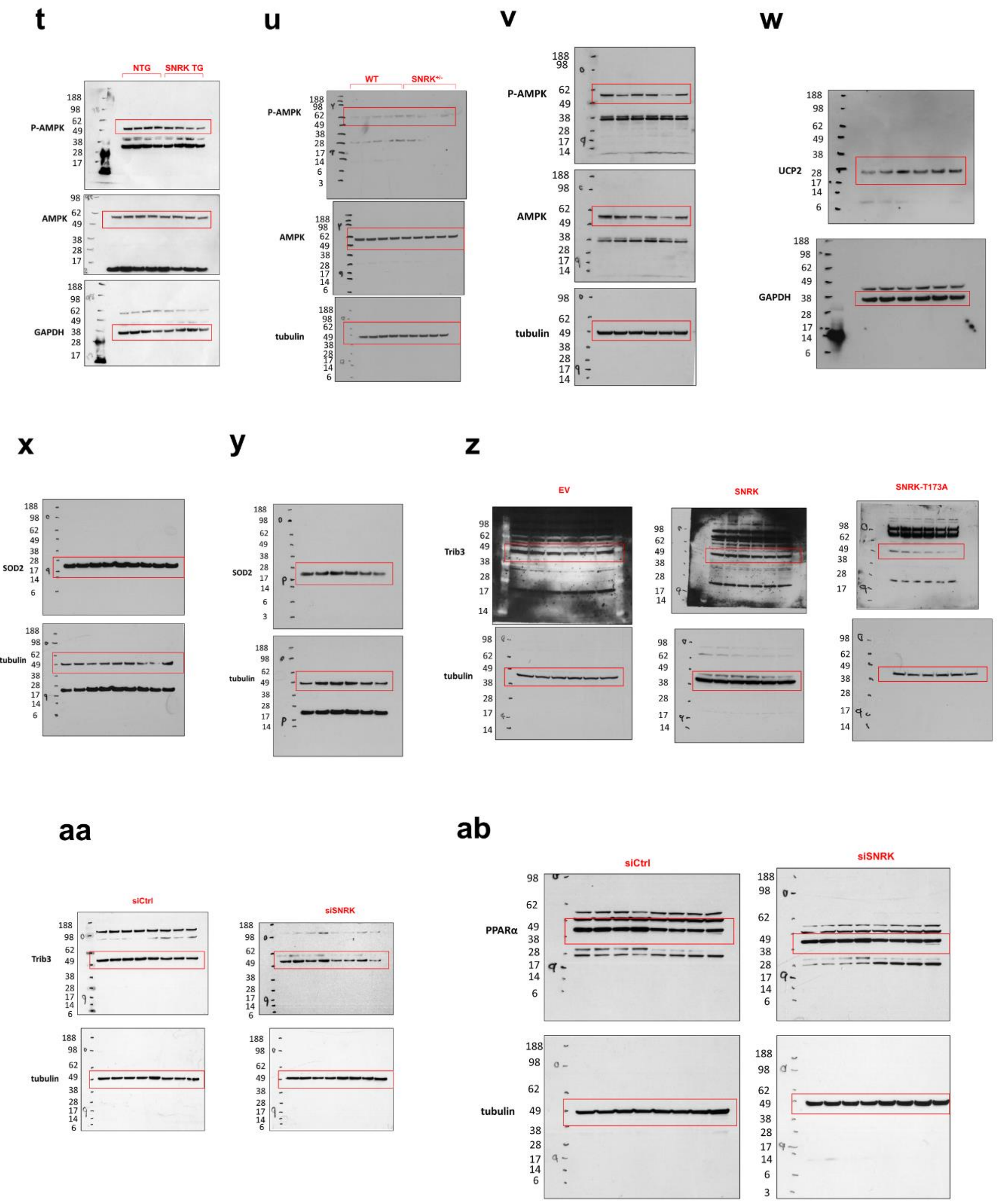

Supplementary Figure 8: Unedited Western blots. Uncut blots for (**t**) Supplemental Fig. 2a, (**u**) Supplemental Fig. 2b, (**v**) Supplemental Fig. 2e, (**w**) Supplemental Fig. 2f, (**x**) Supplemental Fig. 4f, (**y**) Supplemental Fig. 4g, (**z**) Supplemental Fig. 6a, (**aa**) Supplemental Fig. 6b, and (**ab**) Supplemental Fig. 6c.

Supplementary Table 1

	Body weight (g)	Wet heart weight (mg)	Dry heart weight (mg)	Heart rate (beats/min)	Aortic SP (mm Hg)
**NTG**	22.9±0.43	136±3.48	26.8±0.90	291±6.98	79.9±1.32
**TG**	22.3±0.52	136±4.24	26.6±0.92	286±8.34	79.4±0.97

	Aortic DP (mm Hg)	Aortic DevP (mm Hg)	Cardiac flow ($L/min/m^2$)	Aortic flow ($L/min/m^2$)	Cardiac output (mL/min)
**NTG**	40.8±1.85	39.2±1.96	2.90±0.08	8.53±0.26	11.4±0.29
**TG**	41.8±1.06	37.6±1.15	2.96±0.13	8.57±0.21	11.5±0.25

Supplementary Table 1: Body weight, heart weight, and cardiac functional measurements of mice and hearts used in SNRK TG isolated working heart perfusions. SP = systolic pressure; DP = diastolic pressure; DevP = developed pressure. n=12.

Supplementary Table 2

	IVS;d (mm)	LVID;d (mm)	LVPW;d (mm)	IVS;s (mm)	LVID;s (mm)	LVPW;s (mm)	LV Vol;d (µL)	LV Vol;s (µL)
**NTG**	0.76±0.02	3.84±0.08	0.67±0.04	1.16±0.03	2.73±0.09	1.00±0.05	64.5±3.26	29.0±2.40
**TG**	0.76±0.02	3.72±0.07	0.74±0.04	1.12±0.03	2.52±0.08	1.09±0.05	59.3±2.53	23.4±1.86

Supplementary Table 2: Cardiac functional measurements in NTG and SNRK TG mice. IVS = interventricular septum thickness; LVID = left ventricular internal dimension; LVPW = left ventricular posterior wall thickness; LV Vol = left ventricular volume; d = diastolic; s = systolic. n=16-18.

Supplementary Table 3

	Body weight (g)	Wet heart weight (mg)	Dry heart weight (mg)	Heart rate (beats/min)	Aortic SP (mm Hg)	Aortic DevP (mm Hg)
**WT**	29.9±1.1	161±10	48.5±2.8	318±17.8	72.7±1.01	17.8±1.00
Snrk$^{+/-}$	29.6±0.78	155±4.1	50.2±2.7	279±18.9	73.3±1.17	15.9±2.34

Supplementary Table 3: Body weight, heart weight, and cardiac functional measurements of mice and hearts used in *Snrk*$^{+/-}$ isolated working heart perfusions. SP = systolic pressure; DevP = developed pressure. n=4-5.

Supplementary Table 4

	IVS;d (mm)	LVID;d (mm)	LVPW;d (mm)	IVS;s (mm)	LVID;s (mm)	LVPW;s (mm)	LV Vol;d (μL)	LV Vol;s (μL)
**WT**	0.68±0.02	3.62±0.16	0.55±0.03	0.84±0.03	2.70±0.24	0.76±0.08	58.7±6.17	24.7±3.18
Snrk $^{+/-}$	0.66±0.05	3.62±0.01	0.53±0.16	0.84±0.06	2.76±0.09	0.94±0.08	54.3±2.33	21.0±1.00

Supplementary Table 4: Cardiac functional measurements in WT and *Snrk*$^{+/-}$ mice. IVS = interventricular septum thickness; LVID = left ventricular internal dimension; LVPW = left ventricular posterior wall thickness; LV Vol = left ventricular volume. n=3.

Supplementary Table 5

	IVS;d (mm)	LVID;d (mm)	LVPW;d (mm)	IVS;s (mm)	LVID;s (mm)	LVPW;s (mm)	LV Vol;d (μl)	LV Vol;s (μl)
**WT**	0.64±0.04	3.96±0.10	0.62±0.02	1.05±0.04	2.67±0.09	0.99±0.05	26.96±1.80	68.34±3.23
**cs*Snrk* KO**	0.73±0.04	3.84±0.13	0.67±0.03	1.09±0.07	2.57±0.10	1.04±0.03	26.33±2.41	67.38±5.44

Supplementary Table 5: Cardiac functional measurements in WT and cs*Snrk* KO mice. IVS = interventricular septum thickness; LVID = left ventricular internal dimension; LVPW = left ventricular posterior wall thickness; LV Vol = left ventricular volume. n=6-10.

Supplementary Table 6

	Body weight (g)	Wet heart weight (mg)	Dry heart weight (mg)	Heart rate (beats/min)	Aortic SP (mm Hg)	Aortic DevP (mm Hg)
**WT**	25.72±0.45	144.1±3.9	34.7±1.0	294±15.6	71.0±1.3	24.5±1.1
**cs*Snrk* KO**	24.87±0.80	142.6±2.9	34±1.1	293±11	68.7±1.2*	18.3±1.4*

Supplementary Table 6: Body weight, heart weight, and cardiac functional measurements of mice and hearts used in cs*Snrk* KO isolated working heart perfusions. SP = systolic pressure; DevP = developed pressure. n=6 for WT, n=10 for cs*Snrk* KO. **P* < 0.05 by Student's t-test.
